# Regenerative Potential of Extracellular Vesicles on Intervertebral Disc Degeneration: What is the EV‐idence?

**DOI:** 10.1002/jsp2.70149

**Published:** 2025-12-25

**Authors:** Daniele Corraini, Chantal Voskamp, Marca H. M. Wauben, Marianna A. Tryfonidou

**Affiliations:** ^1^ Department of Clinical Sciences Faculty of Veterinary Medicine, Utrecht University Utrecht the Netherlands; ^2^ Regenerative Medicine Centre Utrecht Utrecht the Netherlands; ^3^ Department of Biomolecular Health Sciences Faculty of Veterinary Medicine, Utrecht University Utrecht the Netherlands

**Keywords:** annulus fibrosus, conditioned medium, exosomes, low back pain, mesenchymal stem cells, microvesicles, notochordal cells, nucleus pulposus, regeneration, therapeutical effect

## Abstract

**Background:**

Extracellular vesicles (EVs) represent a promising cell‐free regenerative therapy. Deciphering their mode and mechanism of action comes with technical and biological challenges. This scoping review presents a complementary perspective to reviews that synthesized EV therapeutic strategies in intervertebral disc (IVD) degeneration. It intends to create awareness of the minimal experimental requirements defined by the International Society for Extracellular Vesicles (MISEV). These have been established to facilitate robust and reproducible protocols in the rapidly expanding EV field, aiming to allow comparative studies, improve reproducibility, and interpretability.

**Methods:**

The MISEV were tailored with IVD‐related considerations. Within the timeframe 2016–2025, 129 articles studying EVs in the context of IVD were identified on PubMed. From each article, experimental information on EV isolation and characterization studies, and on functional studies was reviewed for compliance to the IVD‐tailored MISEV.

**Results:**

The information reporting rates were 57% for EV characterization studies and 67% for EV functional studies. Information on EV nomenclature, storage, quantification, and methodological controls for functional studies specifically needs better reporting. Most studies explore EV functionality through conditioned media processed to be enriched for EVs. These EV‐enriched media represent the secretome, the entire collection of secreted molecules, including EVs and co‐isolates. In functional studies, intending to study biological effects driven by the EV‐cargo, inclusion of key methodological MISEV controls is essential.

**Conclusions:**

Here, we sketch and discuss the consolidated “EV‐idence” of EV‐mediated IVD tissue regeneration with studies that have specifically included the minimal MISEV requirements, i.e., (1) EV‐depletion of serum when supplemented in culture medium and (2) inclusion of EV‐depleted conditioned medium as a negative control. Although in several studies, EVs showed homeostatic effects and halted IVD degeneration, solid conclusions are constrained by the limited number of studies complying with the MISEV guidelines.

## Graphical abstract

Summary of the considerations for EV research in the intervertebral disc (IVD) field. Blue text = critical for IVD field when conducting EV functional studies; bold blue exclamation mark = minimal MISEV requirements. EV, Extracellular vesicles; cryo‐EM, cryo‐electron microscopy; hrFC, high‐resolution flow cytometry; MRI, magnetic resonance imaging; NTA, nanoparticle tracking analysis; PCR, polymerase chain reaction; TEM, transmission electron microscopy; WB, western blot.

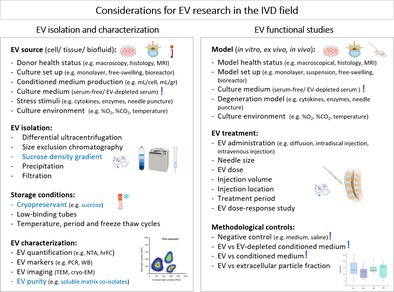

## 1 Introduction

Low back pain (LBP) affects more than half a million people and represents the leading cause of years lived in disability worldwide [1]. LBP has been associated with intervertebral disc (IVD) degeneration based on clinical studies [2, 3] and system‐dynamics methodology [4]. Currently, there is no curative treatment, and the approved treatments developed for intra‐discal application temporarily relieve the pain, causing disability without consistently demonstrating improvement at the IVD tissue level [5]. For LBP patients with advanced IVD degeneration and when conservative treatments fail, surgical interventions are considered, but their effectiveness is widely debated [6]. This highlights the urgency for the development of innovative regenerative approaches that have the potency to address the underlying degenerative processes at an earlier stage.

To mitigate and reverse the pathophysiology of IVD degeneration, different novel therapeutical approaches, either cell‐based or cell‐free, such as cell transplantation, biomaterials, and nanoparticles, are being explored and have been reviewed by Binch et al. (2021) [7]. However, they all present strengths and limitations due to the harsh environment and the avascular nature of the IVD [8]. Among the innovative regenerative approaches, extracellular vesicles (EVs) have emerged as a promising regenerative cell‐free therapy. EVs are non‐replicable cell‐derived membrane‐surrounded small vesicles that are released by all cell types. They are key mediators in intercellular communication and homeostatic regulation by transporting bioactive factors, such as proteins, nucleic acids, and metabolites to recipient cells. Therefore, EVs have the advantage of overcoming the limitations of cell‐based therapies [9], like poor cell viability and bioethical issues that arise from using human‐sourced cell products in allogeneic applications [10].

EVs are increasingly studied as a cell‐derived therapeutic approach within the field of IVD regeneration. Reviews highlight that EV‐based approaches reduce cell apoptosis, inflammation, and extracellular matrix (ECM) catabolism in NP cells in vitro, and retard IVD structure loss, including the preservation of disc height upon IVD degeneration induction in vivo. Therapeutic approaches use source biofluids or tissue and cells unstimulated or cultured under specific conditions (e.g., cytokines, hypoxia, pH) with the aim to improve the yield and the biological efficacy [11, 12]. Furthermore, there is an increasing body of literature where cells are genetically engineered to overexpress bioactive factors (e.g., proteins, miRNAs), with the ultimate aim of enriching the secretome with the target biomolecule eliciting regenerative effects [13]. In both approaches, conditioned media are collected and EVs are enriched and then tested. Such formulations represent, in essence, an EV‐enriched secretome containing EVs as well as co‐isolated biomolecules, like non‐EV‐associated proteins, lipids, and nucleic acids. The treatment is systemically or intradiscally administered, with the latter overcoming targeting issues and decreasing off‐target effects.

EV‐research faces biological and technical challenges defining the EV‐mediated mode and mechanism of action, thereby hampering the translation to the clinic. EVs are highly heterogeneous and are often roughly classified based on their biogenesis route, e.g., exosomes deriving from the endosomal route and ectosomes or microvesicles originating at the cell plasma membrane, or based on their size, such as small or large EVs [14]. Exosomes are typically defined as small EVs (between 30 and 150 nm in diameter), while plasma membrane‐derived EVs have a much larger size range from approximately 30 nm up to several microns, e.g., large oncosomes derived from tumor cells and apoptotic bodies arising from programmed cell death [14, 15, 16]. Even though all these EV subsets are derived from different cellular processes, there are currently no robustly defined EV markers that could distinguish the different EV subsets, once released into the environment [14, 15, 16]. Another complicating factor is the presence of colloidal biological structures, e.g., lipoprotein particles, with partially overlapping physicochemical characteristics with EVs that can be co‐isolated. This requires extensive EV characterization and proper methodological controls to claim an EV‐mediated effect [14, 16].

To address challenges in EV research, the International Society of Extracellular Vesicles (ISEV) defined minimal experimental and information reporting requirements. ISEV published the first minimal experimental requirements (MISEV guidelines) for EVs definition and their functions in 2014 [17], and in 2018, these were supplemented with requirements for minimal information for in vitro and in vivo EV studies [16]. In 2024, the last update of MISEV guidelines was published, providing an updated overview of methods and recommendations for collection, separation, and characterization of EVs from various sources, along with new sections on EV release and uptake [14]. The exhaustive list of the MISEV guidelines was established to facilitate robust and rigorous EV research, with the ultimate aim to allow for comparative analysis among studies, improve reproducibility, and interpretability of the reported findings. Within this list, a subset of guidelines was defined to ensure evidence‐based conclusions on EV‐mediated effects: “the EV‐idence”.

This scoping review presents a complementary perspective to the literature reviews [10, 18, 19, 20] that synthesized the functional outcomes of EV‐enriched formulations and their potential application for the treatment of IVD degeneration. Instead, it intends to create awareness of the MISEV guidelines and the relevance of EV‐depleted and procedural controls considered to be crucial when studying EV‐specific effects. To this end, we analyzed the state‐of‐the‐art literature with respect to conducting and reporting the minimal MISEV requirements. To address this in an IVD‐specific approach, the MISEV guidelines were further tailored with IVD‐related considerations, such as disc cell and tissue properties and culture conditions. Based on this analysis, we sketch the consolidated evidence of EV‐mediated IVD tissue regeneration and discuss future perspectives for EV‐idence and its relevance for clinical translation.

## 2 Scoping Literature Review Methodology

A primary literature search was conducted in PubMed on 31st December 2023 to explore the general compliance to the MISEV guidelines in the IVD field [16]. Subsequently, an updated literature search was performed on September 1, 2025, to synthesize the available EV‐idence on functional studies assessing the biological activity of EVs (Section 3.8). Only peer‐reviewed primary research articles available on PubMed and reporting in the English language were included (search query is provided in Supporting Information).

A total of 93 peer‐reviewed articles were identified in the primary literature search, of which 14 articles were excluded (Table S1) because they were reviews or did not concern the IVD field, which led to the inclusion of 79 articles in the compliance with the MISEV guidelines analysis (Sections 3.2, 3.7; Table S2). The summary table from the MISEV guidelines was adapted to collect data and information regarding EV production, isolation, characterization, and EV testing on IVD cells or tissues (Tables 1 and S3) [16].

**TABLE 1:** Summary of the information categories for EV studies in the intervertebral disc field according to the tailored MISEV guidelines.

	Macro‐categories	Information categories		
EV characterization studies	Nomenclature	(*EVs, exosomes, microvesicles, apoptotic bodies*)		
	EV source	*Cell culture*	*Biofluids or tissue culture*	
	*Donor characteristics*	Donor species (*incl. strain for rodents; breed for large animals*)		
		Donor sex		
		Donor age		
		Donor cell type	Sample type (*tissue, biofluid*)	
		Donor health status (e.g., *macroscopical, histology, MRI*)		
		Cell culture passage	—	
		Mycoplasma test (*cell line*)	—	
	*Culture conditions*	Culture medium composition (*additives, serum, other*)		
		Medium contaminants depletion protocol (*FBS, plasma, natural matrix*)^a^		
		Culture medium volume (*cells/mL*)	Collected CM (mL/g) or biofluid volume	
		Cell density (*cells/cm* ^*2*^)	—	
		—	Collection site (*sampling location*)	
		—	Time and temperature of biofluid or tissue collection (*fresh, cadaveric*)	
		—	Total pooled sample volume or tissue mass	
		Live/dead cells (%)		
		Culture period		
		CM harvest frequency	CM or biofluid harvest frequency	
		Cell treatment (*stimuli*)	Sample treatment (*stimuli*, e.g., *cytokines*)	
		Cell culture setup (*monolayer, 3D, suspension*)	Tissue culture set up (*free swelling, bioreactor, spinner flask*)	
		Culture environment (*% O*_*2*_, *% CO*_*2*_, *temperature*)		
		—	Tissue treatment method for direct EVs isolation (*enzymatic activity or mg/mL, chemical–physical*)	
		—	Treatment time	
		—	Treatment temperature	
	Biofluids, CM, and EV storage	CM or biofluid or tissue storage temperature		
		Storage vessel (*low‐binding tubes*)		
		EVs storage method (e.g., *frozen, lyophilized, sucrose*)		
		EV storage vessel (*low‐binding tubes*)		
		EVs storage temperature		
	EV isolation and concentration	EV isolation method (*ultracentrifugation, density gradient, chromatography, precipitation, filtration, antibody‐affinity*)		
		Detailed EV isolation protocol		
		Concentration method (e.g., *centrifugal filter tube, TFF*)		
		Matrix or sample washing method		
		High recovery/high purity, low recovery/high purity, high recovery/low purity, low recovery/low purity		
	EV quantification	Fluid volume or cell number or tissue mass used to isolate EVs		
		EVs number/mass or volume or cell number		
		Protein amount/mass or volume or cell number (*co‐isolates*)		
		Lipid amount/mass or volume or cell number		
		Ratio of 2 quantification methods (*normalize for protein co‐isolated*)		
	Bulk EV characterization	Marker detection method (e.g., *qPCR, WB, FC*)		
		Transmembrane or GPI‐anchored protein localized in cells at plasma membrane or endosomes		
		Cytosolic protein with membrane‐binding		
		Cytosolic protein with association capacity (*co‐isolated structure*)		
	Single EV characterization	EVs imaging (*TEM, cryo‐EM*)		
		EVs analysis (e.g., *NTA, TRPS, hrFC*)		
EV functional studies	EV recipient model	In vitro	Ex vivo	In vivo
	*Model characteristics*	Model species (*incl. strain for rodents; breed for large animals*)		
		Model sex		
		Model age		
		Model type		
		Model health status (e.g., *macroscopical, histology, MRI*)		
	*Culture conditions*	Experimental set up (in vitro, ex vivo, in vivo)		
		Cell culture passage	—	—
		Culture medium composition (*additives, serum, other*)		—
		Medium contaminants depletion protocol (*FBS, plasma, natural matrix*)^a^		—
		Cell culture condition (*monolayer, suspension, 3D*)	Tissue culture condition (*free swelling, constrained, bioreactor*)	—
		Cell density (*cell/cm* ^*2*^ *cell/mL*)	—	—
		Degeneration induction model (e.g., *cytokines, needle puncture*)		
		Culture environment (*% O*_*2*_, *% CO*_*2*_, *temperature*)		
	*EV treatment*	—	EVs administration (*diffusion, intradiscal injection*)	EVs administration (*intradiscal injection, intravenous injection*)
		EV amount		
		EV treatment period		
	Methodological controls	Dose–response study		
		Negative control inclusion (e.g., medium)^a^		
		EV vs. EV‐depleted CM vs. CM study^a^		
		EV vs. EP study		
	Analysis	Gene/protein expression, biochemical/histological/radiological/biomechanical analysis, pain assessment		

*Note:* The MISEV 2018 guidelines were tailored to integrate additional categories relevant to the intervertebral disc (IVD) field and were used to collect the information from the 79 selected peer‐reviewed articles.

Abbreviations: CM, conditioned medium; cryo‐EM, cryo‐electron microscopy; EP, extracellular proteins; EV, extracellular vesicles; FBS, fetal bovine serum; FC, flow cytometry; hrFC, high‐resolution flow cytometry; MRI, Magnetic resonance imaging; NTA, nanoparticle tracking analysis; qPCR, quantitative polymerase chain reaction; TEM, transmission electron microscopy; TFF, tangent flow filtration; TRPS, tuneable resistive pulse sensing; WB, western blot.

^a^ Indicates minimal MISEV requirements.

**FIGURE 1:**
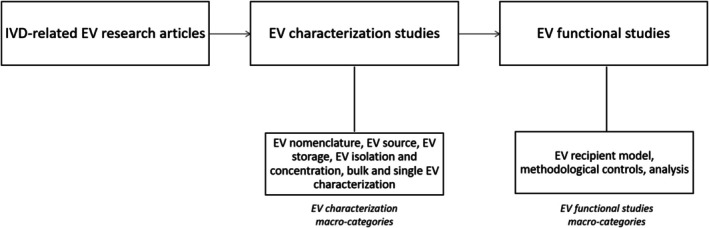
Schematic representation of data retrieval flow. Macro‐categories information is provided in Table 1.

In each primary research article, every EV source used was defined as a separate EV characterization study. Moreover, in the case where the isolated EVs were further tested on different models, each testing model was defined as an individual EV functional study. For each study, reporting data were collected (Figure 1) and systematically analyzed following the respective information categories of Table 1, without assessing the completeness, rigor, or quality of the reported information. Missing data were indicated as not reported. In the event that specific information categories were not addressed because of the experimental setup or research question, they were indicated as not applicable.

The reporting percentage of each study was determined by the number of reported information categories out of the total number of applicable categories. Next, reporting percentages were calculated for each category (the number of studies that reported such information out of the total number of studies applicable for the respective information category), and these were subsequently averaged to generate scores for corresponding macro‐categories. Finally, these macro‐categories were combined to calculate a final overall reporting percentage of EV characterization studies and EV functional studies. This altogether resulted in a matrix of the information reporting status of the literature regarding EV studies in the IVD field (Table 1, Figure 1).

For the EV‐idence analysis, a total of 53 new articles were identified from the secondary literature search; however, 3 articles were excluded due to being off‐topic or review papers (Tables S4 and S5). Altogether, 129 peer‐reviewed articles were reviewed to synthesize EV‐specific functional effects (Section 3.8).

## 3 Results

### 3.1 EV Studies in the IVD Field Included in This Scoping Review

**FIGURE 2:**
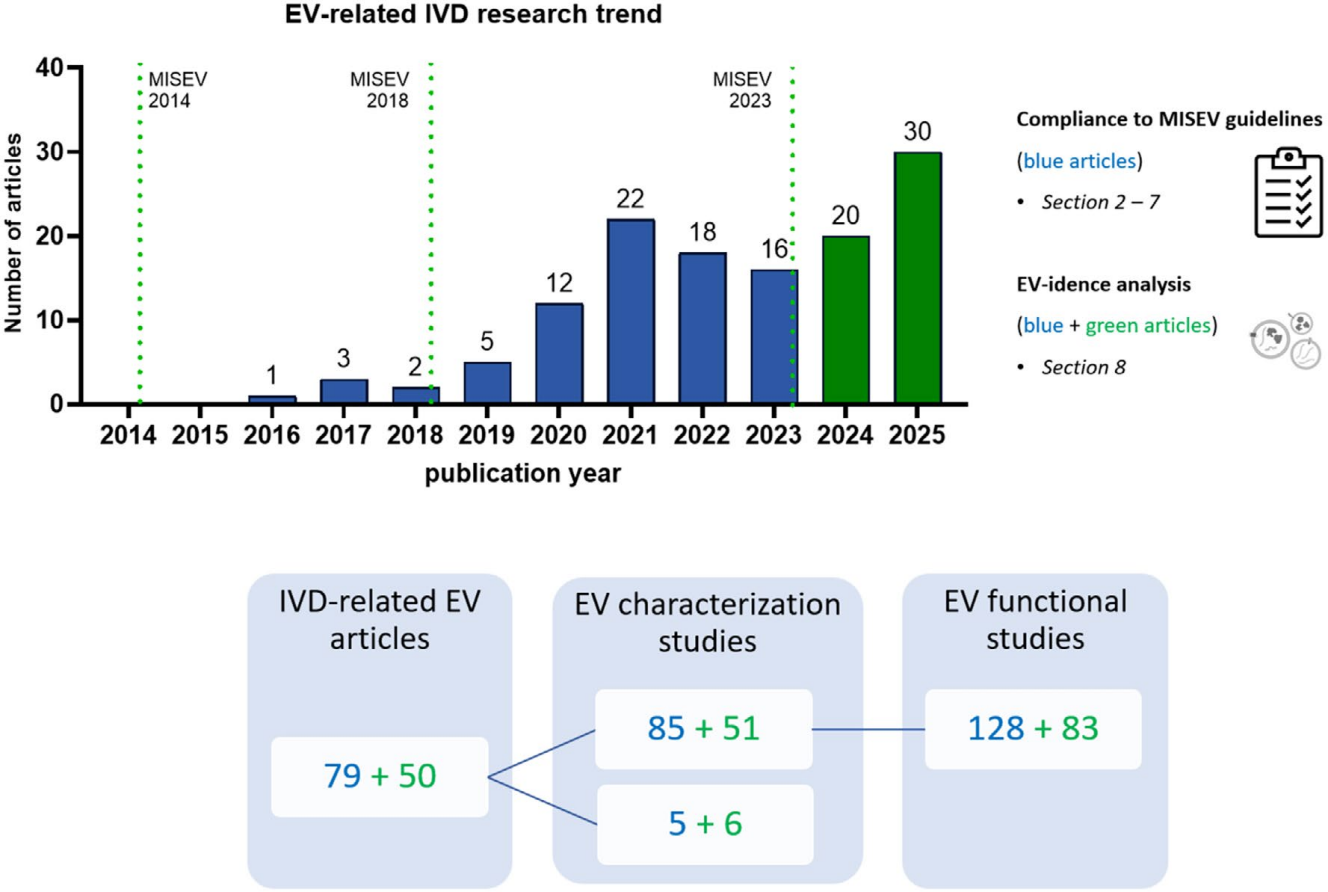
Distribution of the EV‐related peer‐reviewed articles in the intervertebral disc (IVD) field. Relevant time points are indicated: The Minimal Guidelines by the International Society for Extracellular Vesicles (MISEV) [14, 16, 17]. The primary literature data set (blue bars; 2016–2023) was used for the experimental information reporting analysis (Sections 3.2, 3.7). The updated literature data set (green bars; 2024–2025) was integrated to the primary data set and used for the EV‐idence analysis (Section 3.8). These articles concern EV characterization and EV functional studies; one article may comprise more than one study on EVs. EV characterization studies may perform EV functional studies on more models ending up with more than one EV functional studies per EV characterization studies.

The first report on EVs in the IVD field was published in 2016, with a steady increase in number since then (Figure 2). Hence, EV research in the IVD field is at its infancy with a growing trend, indicating its potential.

For the information reporting analysis (Sections 3.2, 3.7), assessing how the field complies with the MISEV nomenclature and guidelines, 79 peer‐reviewed articles spanning from 2016 to 2023 were analyzed (Figure 2). Several articles comprised more than one study in which EVs were produced from various sources or under different conditions, resulting in a total of 90 studies on EV production and characterization. Moreover, 85 of these studies further tested the isolated EVs in one or more different functional models, resulting in 128 EV functional studies (Figure 2). In total, including the 5 EV characterization studies, 133 EV studies were systematically scored for reporting the information of the distinct categories as indicated in Table 1. The updated literature search spanning between 2024 and 2025 retrieved 50 additional articles, comprising 83 additional EV functional studies, that were included on top of the previously identified 128 studies, for an up‐to‐date EV‐idence analysis reported in Section 3.8 (Figure 2).

### 3.2 EV Nomenclature

Across different research fields, EVs have been traditionally referred to with biogenesis‐related nomenclature such as exosomes, microvesicles, and apoptotic bodies. However, as highlighted by the ISEV, this historical nomenclature cannot be used accurately, particularly as it remains difficult to prove the differential intracellular origin of isolated EVs due to the absence of a consensus on specific molecular markers for the different EV subtypes [21]. Acknowledging this bottleneck, the ISEV determined “extracellular vesicles” (EVs) as the preferred nomenclature for cell‐released particles that are devoid of a functional nucleus, delimited by a lipid bilayer, and are unable to replicate. The ISEV discourages the use of biogenesis‐based terms unless such an EV population is caught in the act of being released by live imaging techniques [16].

**FIGURE 3:**
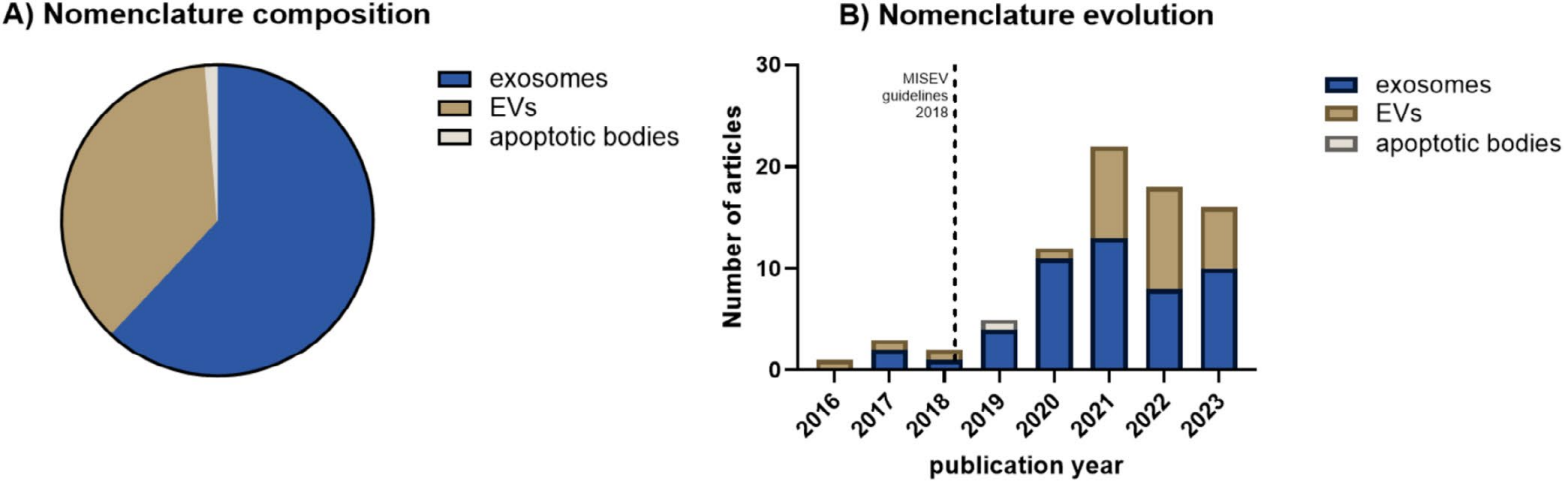
Nomenclature of cell‐derived vesicles in the IVD field. Pie chart (A) and stack histogram (B) showing the EV nomenclature in the 79 peer‐reviewed studies (2016–2023) that were analyzed with respect to reporting information following the MISEV nomenclature.

The reporting analysis showed that 36.7% of the articles used the preferred term “extracellular vesicles,” while the majority used the term exosomes (62.0%) without proving their origin from multivesicular bodies (Figure 3A). One article referred to the term apoptotic bodies in a study with particles that were generated on oxidative stress, which caused cell death [22]. These particles were isolated by differential ultracentrifugation and filtration and tested for the apoptotic body marker histone 3 expression. Although our analysis of the nomenclature used in the IVD field showed heterogeneous terminology over 2016–2023, an increasing, yet incomplete, adoption of the EV nomenclature of the 2018 MISEV guidelines is observed (Figure 3B).

### 3.3 EV Source, CM, and EV Storage

In EV studies, the first step entails the production or collection of an EV‐containing source, such as conditioned medium from cell or tissue culture, or biofluids. During this step, several factors may have an influence and require detailed reporting of methodology, including the EV source characteristics, material handling, such as collection, pre‐processing, and storage.

#### 3.3.1 EV Source

**FIGURE 4:**
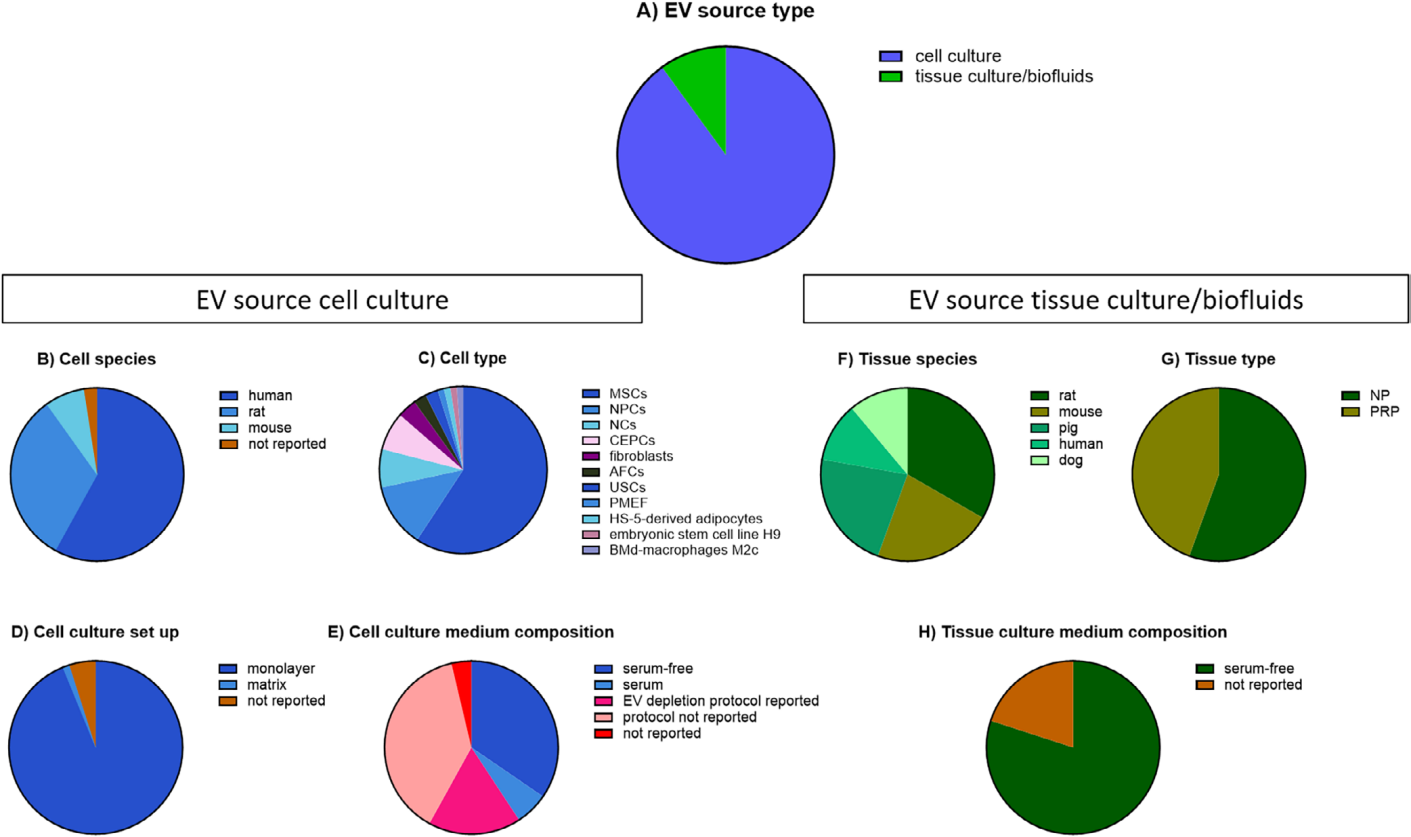
Overview of EV source in the IVD field. (A) Pie chart of the 90 applicable studies showing from which sources EVs were produced covering the years 2016–2023. Pie chart of 81 applicable studies where EVs derived from cell culture showing (B) cell species, (C) cell type, (D) cell culture set‐up, and (E) cell culture medium composition. Pie chart of 9 applicable studies where EVs derived from tissue culture/biofluids showing (F) tissue species, (G) tissue type, and (H) tissue culture medium composition for explant culture. Mesenchymal stem cells (MSCs), nucleus pulposus cells (NPCs), cartilaginous end plate cells (CEPCs), annulus fibrosus cells (AFCs), umbilical stem cells (USCs), notochordal cells (NCs), primary mouse embryonic fibroblast (PMEF), bone marrow‐derived (BMd‐) macrophages, nucleus pulposus (NP), platelet‐rich plasma (PRP).

All 90 studies, in which EVs were produced and characterized, reported the information regarding the EVs' source type (Figure 4A). In most of the studies (*n* = 81), EVs were derived from cultured cells, either primary cells or cell lines (Table S6). Only 9 studies used EVs derived from tissue cultures or liquid tissue/biofluids (Table S7).

Categories with an average score above 95% were donor cell/tissue species and type, cell/tissue treatment and culture condition, and cell culture medium composition. Other categories were less well reported, such as the sex origin of cells or tissues (36% and 44%, respectively), the protocol to deplete contaminants, including EV depletion from cell or tissue culture media supplemented with serum (29% and 0%, respectively), and cell viability in culture (5%) or within the cultured tissue (0%). In addition, the information categories specific for cell culture that were scarcely reported were donor age of cell origin, referring to the age of the donor who provided the cells, mycoplasma test on cell lines (6%), and the cell culture medium volume used to generate EVs (17%). Less well‐reported categories specific for tissue culture were the total pooled volume of biofluids or grams of tissue used to generate EVs (44%). Detailed documentation of the EV‐source methodology is pertinent to the IVD field. We highlight below how the role of donor species and type, culture set up, and culture medium composition could influence EV production, characteristics, and ultimately their functional properties.

EVs originating from different cell and tissue sources can exhibit different characteristics and molecular cargo, reflecting their origin, ultimately leading to distinct biological effects [23]. The same applies for the same cell type derived from different species [24, 25, 26]. A well‐known example is the cellular composition of the NP differing among species [27, 28], with IVDs that are notochordal cell‐rich (e.g., rats and pigs) as opposed to IVDs rich in the smaller non‐vacuolated NP cells (e.g., bovine and human). These differences are linked to differences in biology and regenerative potential [29, 30], and might be reflected in the characteristics and function of the EVs they secrete. Recently, changes in the proteomic EV cargo of human IVD cells have been reported across different stages of IVD degeneration [31]. This illustrates the necessity of detailing in the methodology section the tissue source and its health status, the IVD cell type, as well as the species used to generate EVs. Furthermore, the composition of the cell and tissue culture medium, including glucose concentration, pH, oxygen tension, and supplemented growth factors, can affect EV production and their properties since these factors influence the cell metabolism and signaling [32, 33]. In particular, medium components that contain exogenous EVs, such as serum (fetal calve or bovine serum [FBS]) or platelet lysate can contaminate the conditioned medium during the EV production [34, 35] and require, according to MISEV, depletion from exogenous EVs [14, 16]. Better adherence to the MISEV guidelines regarding this aspect will increase the rigor and reproducibility in the EV‐IVD field and enable scientists to better comprehend source‐ and tissue‐dependent EV effects.

##### 3.3.1.1 EV Source Donor Species and Type

In 79 of 81 studies, where EVs were produced from cell culture, the cell donor species and cell type used were indicated (Figure 4B,C). Furthermore, all 9 studies, where EVs were produced from tissue culture, reported the tissue donor species and tissue type (Figure 4F,G). This information highlights that multiple species were used in EV production from cells or tissue, including human (58% for cell source, 11% for tissue source); rat (32% for cell source, 33% for tissue source); mouse (7% for cell source, 22% for tissue source); pig (22% for tissue source); and dog (11% for tissue source) (Figure 4A,F). With respect to the cultured cell type, both primary cells (63 studies) and cell lines (18 studies) were used. The majority (48 out of 81 studies) used mesenchymal stromal cells (MSCs) as EV source with a translation potential as a cell‐free regenerative therapy (Figure 4C). With respect to IVD cells, EVs were generated from culturing cells of the nucleus pulposus, in specific, small non‐vacuolated nucleus pulposus cells (NPCs; 10 studies) or vacuolated notochordal cells (NCs; 6 studies); however, the latter notably lose their vacuolated phenotype when cultured and expanded [36]. Furthermore, EVs were generated from annulus fibrosus cells (AFCs; 2 studies) and the cartilaginous endplate, cartilaginous endplate cells (CEPCs; 6 studies) (Figure 4C). Moreover, 3 studies used fibroblasts, the main cell type of connective tissue, but absent in the healthy IVD, as a control to test the cell type‐specificity of EVs [37] (Figure 4C). Regarding the tissue type, 56% were derived from NP explant cultures and 44% were isolated from platelet‐rich plasma (PRP) (Figure 4G). Altogether, it is notable that in the IVD field, the majority of the EVs are derived from primary cells, while 18 studies used cell lines as EV source.

##### 3.3.1.2 EV Source Culture Set Up

The cell culture set up, such as monolayer, suspension, or 3D cell culture, can affect the EV production and release, concentration, and their functional properties [38, 39]. Of 81 studies, 77 studies reported such information. Most of the studies (*n* = 76) produced EVs in monolayer cell culture, while 1 study cultured cells on a matrix substrate made of Matrigel (Figure 4D). Matrigel is the biological solubilized basement membrane matrix produced by Engelbreth‐Holm‐Swarm (EHS) mouse sarcoma cells [40], which might contain EVs, as also described for other biological‐derived cell culture products. Furthermore, during EV production, EVs may interact with the Matrigel via (covalent) binding between EV‐associated proteins and ECM proteins, thereby affecting the bioavailability of the EVs [41]. As the IVD field moves to in vivo‐like culture conditions with the use of naturally derived biomaterials, these notions are crucial to consider together with appropriate controls (Figure 4D).

Likewise, the EV production from tissue culture is influenced by the conditions in which the explant is cultured, such as free swelling, bioreactor, or spinner flask [23]. This does not apply for direct isolation of EVs from liquid tissue like blood plasma (4 of 9 studies). All five explant culture studies cultured tissue explants in free swelling conditions to generate EVs, and all of them reported the relevant information. In this respect, NP explants are differentially affected under free swelling or constrained conditions, leading to a distinct biologic response of the resident cells [42] and possibly their secreted EVs.

##### 3.3.1.3 EV Source Culture Medium

Medium composition used during EV production by means of cell culture was reported in 78 of 81 studies, of which 35% used serum‐free media, while 62% used media containing serum. Most of the latter studies depleted the serum from exogenous contaminating EVs (45 of 50 studies), which is fundamental in avoiding unrelated effects of exogenous serum‐derived EVs. Of those, only 31% reported the protocol used for EV depletion from the serum (Figure 4E). Only one tissue culture study out of 5 did not report the medium composition, while all other studies report the use of serum‐free medium (Figure 4H). Overall, reporting information of culture media used to generate EVs is well reported, while detailed reporting of the depletion method of the serum is needed to allow evaluation of the EV depletion efficacy and the EV‐mediated effect.

#### 3.3.2 Biofluids, CM, and EV Storage

The ISEV has thoroughly addressed how storage conditions of biofluids, conditioned medium, and isolated EVs pelleted or resuspended in medium or PBS affect EV characteristics and properties, such as stability, concentration, yields, aggregation, content, and function [14, 16]. In particular, the concentration of EVs has been shown to decrease when regular storage vessels are used because EVs attach to their surface; therefore, low‐binding vessels are recommended [43]. Moreover, the concentration of EVs and their functionality are reported to decrease upon freeze–thaw cycles, and hence it is suggested to minimize the number of freeze–thaw cycles [44, 45]. Importantly, cryoprotectants, such as sucrose or trehalose, used to reduce the influence of freeze–thawing cycles on EVs' integrity [46], are difficult to remove from the EV preparation and may have an impact on the biologic effects of EVs. Moreover, especially in the context of the IVD field, this could complicate functional EV studies in the IVD, where the field strives to mimic the disc environment with relatively low glucose media levels [47].

**FIGURE 5:**
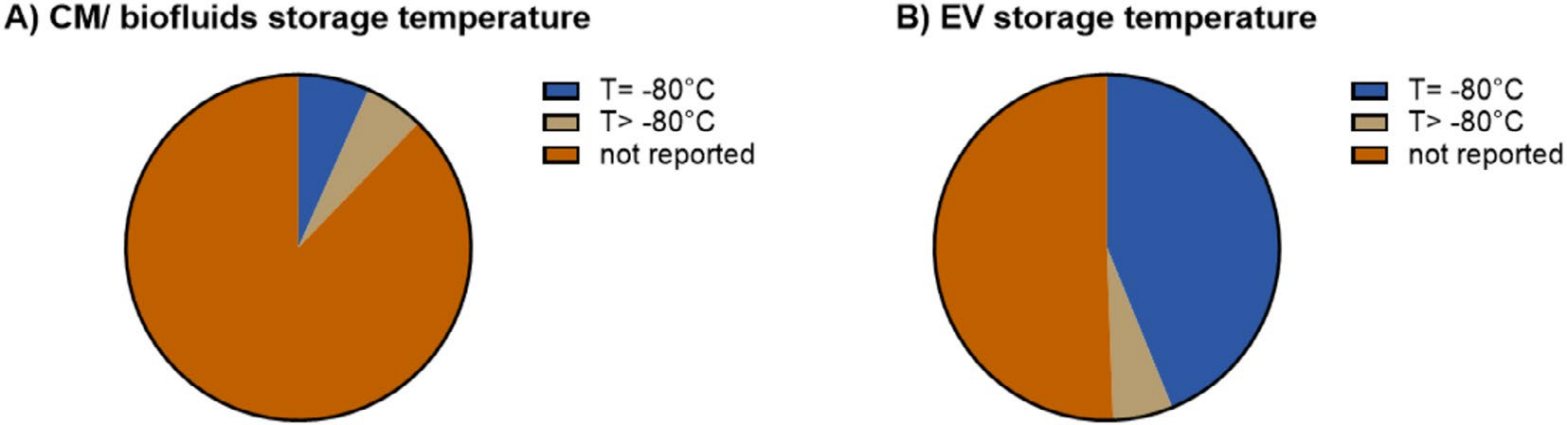
Storage temperature for EV source matrix and isolated EVs. Pie chart of the storage temperature of (A) the biofluids or conditioned medium (CM) in 90 applicable studies and (B) of the isolated EVs in 89 applicable studies covering the years 2016–2023.

While most of the studies (65 of 89) reported the storage method for the EV preparations, further information regarding the storage temperature and vessels of both the tissue source and the isolated EVs was poorly reported (Table S8). Notably, the temperature of storage was reported in 12% of the studies for the collected biofluids or conditioned medium (Figure 5A), and in 49% of the studies for the isolated EVs (Figure 5B). In general, storage of such samples under standard laboratory conditions at −80°C is considered to preserve EVs' integrity and therefore their function [48]. Within the context of biofluids, it is noteworthy that recent study has shown that the stability of EVs stored in plain PBS declines drastically, while their stability was maintained for at least 20 weeks in the presence of bovine serum albumin [45].

Altogether, considerations on storage methods, temperature, and vessels are essential, and the optimal storage conditions for isolated EVs from cells or biofluids need to be defined. It is unlikely that a single universal optimal storage condition exists, as storage conditions are also dependent on the co‐isolates in EV preparations. Co‐isolates vary between different cell cultures and biofluids. Hence, besides the more “general” guidelines on storage vessels and the influence of freeze–thaw cycles, the storage requirements will need to be tailored for EV preparations and their intended use.

### 3.4 EV Isolation and Concentration

EV isolation represents the next step to collect EVs for further analysis and functional studies. Absolute purification of EVs is considered unrealistic, since the source biofluids and conditioned media are colloids and contain extracellular particles with partially overlapping physiochemical properties [49]. As extensively discussed in the MISEV, EV enrichment can be performed with several isolation methods, with their respective levels of enrichment and purity [16, 50]. Furthermore, the isolation method can be eventually combined with sample concentration and EV pellet washing steps, which can also affect the integrity and, therefore, the functional properties of EVs. The respective information on EV isolation and concentration procedures is well reported for EV research in the IVD field (98%; Table S9).

**FIGURE 6:**
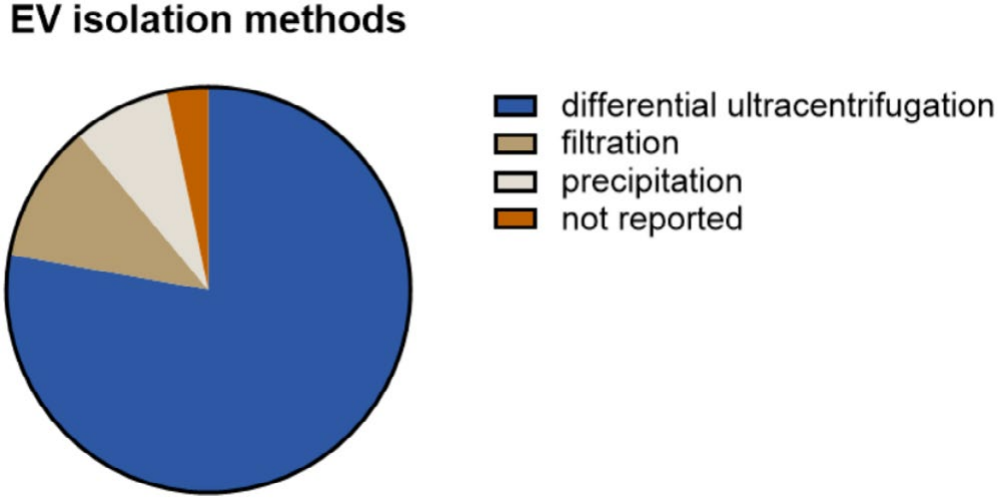
EV isolation methods used in the IVD field. Pie chart of 90 applicable studies from 2016 to 2023 showing the primary isolation method used to enrich for EVs.

The vast majority of the studies reported the EV isolation method, with 86 out of 90 studies using a single method for EV enrichment. Differential ultracentrifugation (78%) was the most common EV enrichment technique used, followed by filtration (11%) and precipitation (8%), while no study used density gradient centrifugation alone (Figure 6). The latter method enriches EVs based on their buoyant density, and for the EV field, it is generally a preferred method in combination with centrifugation and/or size exclusion separation to obtain relatively pure EV preparations, minimizing the number of non‐EV particles inevitably co‐isolated [51]. However, density gradient centrifugation is cumbersome, EV yields are relatively low, and, specifically for the IVD field, density gradient material, e.g., sucrose, poses challenges as it requires additional steps to be removed to prevent the presence of sucrose from interfering in EV functional assays conducted in culture conditions mimicking the low‐glucose disc environment. Nonetheless, density gradient centrifugation is an appropriate isolation method that ensures high EV purity ideal for EV characterization.

Different isolation techniques, with respective recovery and specificity efficiency, can be combined sequentially to further enrich and purify EVs. Four studies used a second method to further enrich for EVs, specifically size exclusion chromatography (1 study) or density gradient (3 studies); in this latter case, bearing the aforementioned challenges for EV functional studies in the IVD field. In fact, in these studies, EVs were not subjected to further EV functional studies (Table S10).

Overall, the EV isolation method is well reported, and the majority of studies used only differential ultracentrifugation to isolate EVs. However, according to the MISEV guidelines, a single isolation method alone is not recommended to be used to show EV‐mediated effects, since extracellular particles are inevitably co‐isolated and could mask the EV‐specific effects. Therefore, a combination of EV enrichment methods is preferred to reduce the presence of co‐isolates and increase EV purity. Further research is required to determine isolation protocols that ensure a combination of the highest EV recovery and purity suitable for IVD samples [14, 16].

### 3.5 EV Quantification and Characterization

On EV isolation, EVs can be quantified for functional dose–response studies and characterized to confirm the presence of EV markers, as well as defining the biological EV‐associated cargo.

#### 3.5.1 EV Quantification

EVs can be quantified with several techniques, such as nanoparticle tracking analysis (NTA), flow cytometry, and resistive pulse sensing (RPS). Other ways to indirectly infer the EVs concentration are represented by the quantification of their components, such as proteins, lipids, nucleic acids, and other biomolecules. However, all these techniques are not specific for EVs, and co‐isolates can hamper quantitative analyses. Each of the techniques, as addressed by the MISEV, has strengths and limitations with respect to particles concentration, size range sensitivity, and EV component analysis [16, 52]. Because of the limitations of each quantification method, the ISEV acknowledges that orthogonal particle analysis, defined by multiple and complementary methods, is recommended to quantify EVs. Reporting of EV quantification information in the IVD field is limited, with an average rate of 14% (Table S11). Conducting and reporting EV concentration‐based experiments could allow for dose‐finding studies in follow‐up functional studies and enable meta‐analysis strengthening evidence‐based preclinical and clinical research in the IVD field.

#### 3.5.2 EV Characterization

EVs present great heterogeneity regarding size, composition, and molecular markers. EV isolates are commonly characterized with EV markers and visualization of single EVs. While the majority of the EV studies in the IVD field report this methodological information, analysis of EV co‐isolated proteins has been less well reported, with an average score of 41% (Table S12). Bulk EV characterization based on EV markers together with markers for co‐isolates, EV imaging, and analysis could provide a better understanding of the relationship between the cell and tissue source and specific EV types. Such characterization is also essential for deciphering truly EV‐mediated effects and for designing appropriate methodological and experimental controls in functional studies.

##### 3.5.2.1 Bulk EV Characterization

**FIGURE 7:**
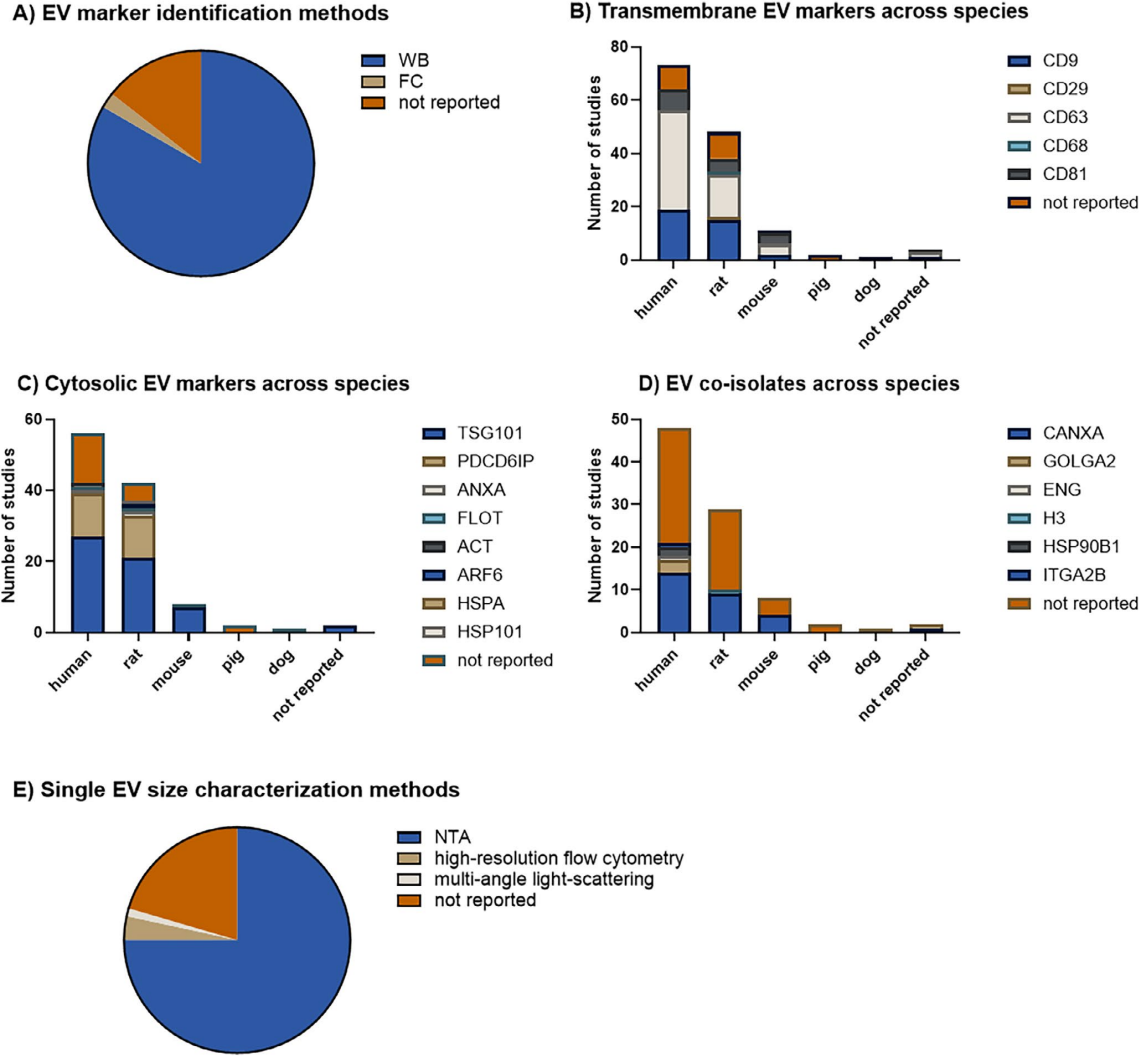
EV characterization. (A) Pie chart of 90 applicable studies from 2016 to 2023 showing the EV marker identification methods used to analyze isolated EVs. Stacked histograms for the transmembrane (B) and cytosolic (C) EV markers and (D) markers for EV purity and biogenesis used across species. Please note that a single study may test multiple markers, resulting in a higher number of markers studied per species compared to the total number of studies per species. (E) Pie chart showing the single EV analysis methods used. ACT, Actin; ANXA, annexin; ARF6, ADP‐ribosylation factor 6; CANXA, calnexin; ENG, endoglin; FC, flow cytometry; FLOT, flotillin; GOLGA2, golgin subfamily A member 2; H3, histone 3; HSPA, heat shock protein A; HSP101, heat shock protein 101; HSP90B1, heat shock protein 90B1; ITGA2B, integrin A2B; NTA, nanoparticle tracking analysis; PDCD6IP, programmed cell death 6‐interacting protein; TSG101, tumor susceptibility gene 101, WB, Western blot.

Despite attempts to define EV subtype‐specific markers [53], specific markers for different types of EVs still remain elusive due to the different (intra)cellular and tissue sources of EVs and the heterogeneity of isolation methods. Therefore, the MISEV guidelines defined two protein marker categories to indicate the presence of EVs in bulk EV preparations, i.e., transmembrane EV markers and cytosolic EV markers. Bulk EV protein characterization is often done via Western blot (WB) or proteomics analysis. The vast majority of EV studies in the IVD field used WB for this purpose (75 of 90 studies), and 2 studies used bead‐based flowcytometric analysis for the analysis of transmembrane EV‐proteins, with only 14% not reporting on such characteristics (Figure 7A). None of the studies further characterized bulk EV via proteomics.

The transmembrane EV markers comprise transmembrane, glycosylphosphatidylinositol (GPI)‐, and lipid‐anchored proteins integrated within the plasmatic and endosomal membranes. They represent a hallmark for all EVs since their presence confirms the lipid‐bilayer structure of the EVs. Among the 90 studies, 67 studies (74%) reported the presence of transmembrane EV markers (Figure 7B). The most reported transmembrane EV markers were the tetraspanin proteins CD9 and CD63. To understand the relation between species and the information reporting rate, we analyzed the distribution of transmembrane EV markers among species (Figure 7B). Since one study may test EVs for multiple markers, this resulted in a higher number of markers per species compared to the number of studies per species. Transmembrane EV markers were reported in 88% of human (73 studies), 79% of rat (48 studies), and 91% of mouse (11 studies), while none of the 2 pig and 1 dog EV studies reported these markers (Figure 7B). The cytosolic EV markers are represented by soluble proteins and lipid‐ or membrane‐anchored proteins comprising the EV cargo. They are an indicator of the structure of any EVs defined by a bi‐layered membrane surrounding intracellular components, either actively incorporated or not. Most of the studies (74%) reported such information (67 of 90 studies) (Figure 7C). The most reported cytosolic EV markers were proteins involved in multivesicular body biogenesis; tumor susceptibility 101 (TSG101) and programmed cell death 6 interacting protein (PDCD6IP, also known as alixin, ALIX) (Figure 7C). These are common cytosolic EV markers transversally analyzed in the EV field. With respect to the studies reporting across species, cytosolic EV markers were reported 75% in human of 56 studies, 88% in rat of 42 studies, 88% in mouse out of 8 studies, while none of the pig (2 studies) and dog (1 study) reported these markers (Figure 7C). For the latter two less‐studied species, the deficient reporting of transmembrane and cytosolic EV markers can be due to the lack of validated and reliable species‐specific antibodies. In this respect, EV markers were recently validated for pigs and dogs using a multiplex‐based WB methodology using notochordal cell‐derived EVs [54]. Despite the availability of validated antibodies for human, rat, and mouse, the lack of reporting in studies in these species might be due to limited availability of starting material, which is not alluded to in the respective studies.

Due to the technical aspects of EV isolation methods, proteins can be co‐isolated with EVs. Such co‐isolated proteins may represent a readout for impurities on EV isolates. However, they can also be part of the EV‐associated corona [55]. Such corona‐associated proteins can be informative, e.g., containing well‐known tissue‐specific markers, or functional proteins, e.g., signaling or ECM proteins [56, 57]. Due to their relevance in EV characterization, reporting information on co‐isolated proteins is crucial. Among the 90 studies, 37 (41%) reported the presence of co‐isolated proteins (Figure 7D). More specifically across species, such information was reported in 44% in human (48 studies), 35% in rat (29 studies), 50% in mouse (8 studies), while none in 2 pig and 1 dog studies (Figure 7D). The most reported co‐isolated protein was calnexin (CANXA), a common origin marker for endoplasmic reticulum and the Golgi apparatus secretory pathway which is mostly absent in EVs (Figure 7D). None of the studies reported co‐isolated proteins specific to the IVD, such as IVD cell markers or ECM components. However, using a multiplex WB‐based technique (Digiwest), the presence of such IVD‐relevant proteins like sonic hedgehog, fibronectin, and integrin beta 1 in notochordal cell‐derived EV preparations was demonstrated [54]. Furthermore, proteomic analyses of cultured human or bovine IVD cells confirmed the presence of ECM components and proteins often associated with non‐EV co‐isolated structures such as apolipoproteins, highlighting the importance of characterizing co‐isolated proteins [31, 58]. Especially, EVs derived from human IVD cells cultured in monolayers and isolated with differential centrifugation followed by size‐exclusion chromatography showed a large number of ECM‐related proteins including numerous glycosaminoglycans, collagens, small leucine‐rich proteoglycans, and laminins, with a different abundance depending on the degree of degeneration [31]. Whether these co‐isolated proteins are derived from co‐isolated contaminants or part of the EV‐corona and contribute to the functional modulation by EVs remains unknown. Reporting on and studying co‐isolated proteins could enhance our understanding in this area.

In summary, appropriate EV marker characterization is required to indicate EV enrichment and to confirm successful EV depletion in EV‐depleted control preparations. In the latter, EV‐related markers are expected to be drastically low as an outcome of such analysis. Moreover, it could allow for better prediction of EV function based on their composition. Reporting on co‐isolated proteins is informative to define possible EV corona components or to identify co‐isolated contaminants.

##### 3.5.2.2 Single EV Characterization

Whereas phenotypic analysis of EVs is often performed on bulk EV preparations, single EV‐based high‐resolution analysis is needed for EV quantification and (bio)physical EV analysis. In regard to EV quantification and size distribution analysis, the majority of the studies (80%, 72 studies) reported such information, with nanoparticle tracking analysis (NTA) being the main technique used (73%; Figure 7E). Regarding EV visualization, 90% of the studies reported having used electron microscopic analysis, demonstrating the presence of lipid bilayer‐enclosed EVs (Table S12).

EV quantification is necessary to determine the EV generation capacity of the EV source under specific conditions, and for dosage finding for EV functional studies. Furthermore, single EV visualization provides insights into the EV morphology and confirms the presence of EVs. However, none of these analyses and characterization methods provide information on the biological effect or functionality of the isolated EVs.

### 3.6 EV Functional Studies

As a new and promising cell‐free therapeutic strategy for IVD degeneration, EVs have been investigated in models with different levels of complexity, including IVD cells, IVD explants, or in animal models, encompassing a cumulative total of 128 functional studies over 2016–2023. Each experimental set up, whether in vitro, ex vivo, or in vivo, has specific procedural and experimental parameters that can influence the outcome of the EV functionality.

#### 3.6.1 EV Functional Study Models

**FIGURE 8:**
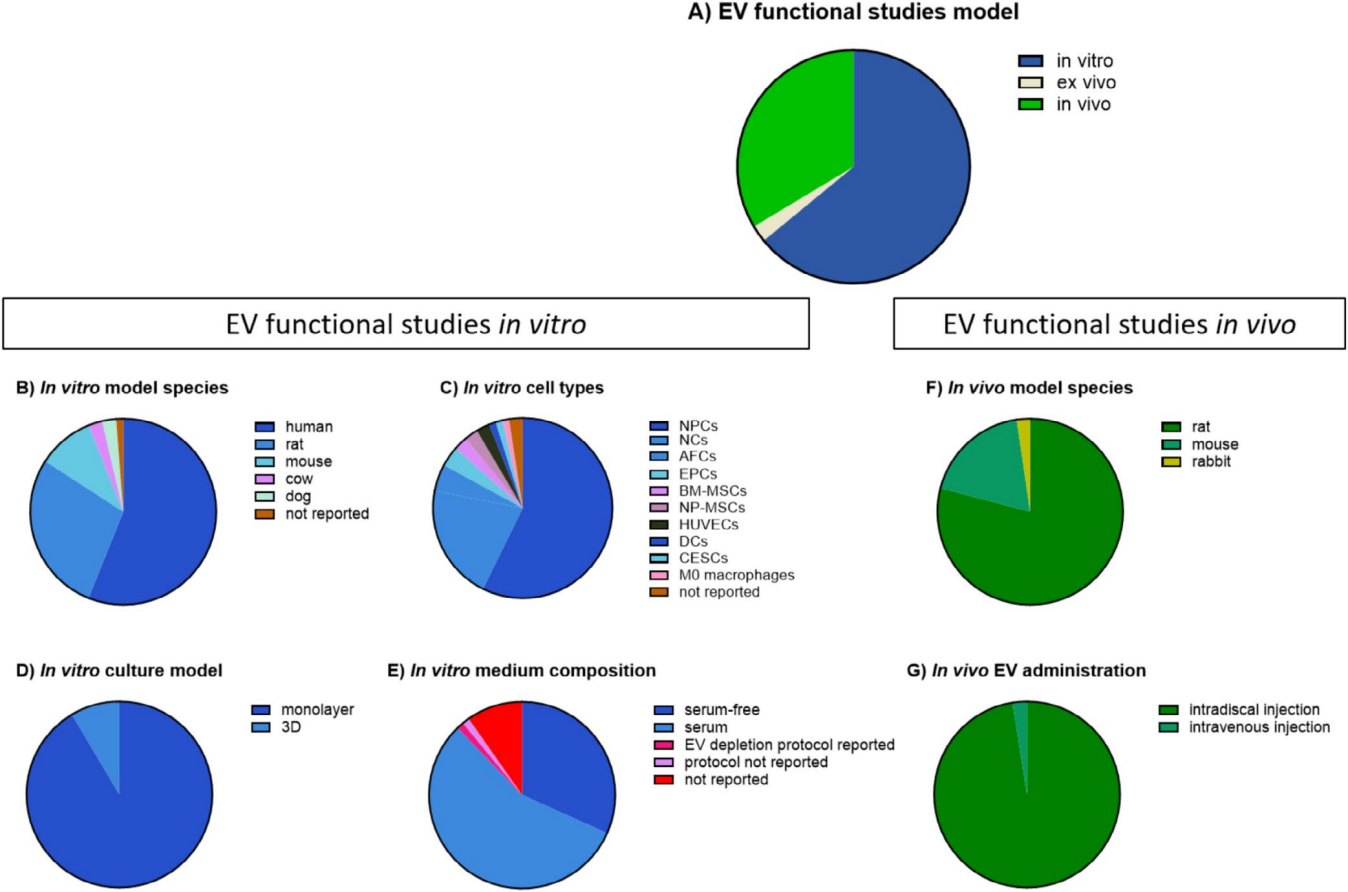
Overview of EV functional studies model in the IVD field. There were 128 applicable studies spanning the period of 2016–2023. (A) Pie chart showing model set‐up. Pie chart of 82 applicable in vitro studies showing (B) cell donor species, (C) cell type, (D) cell culture model, and (E) cell culture medium composition. Pie chart of 43 applicable in vivo studies showing (F) model species and (G) way of EV administration. Nucleus pulposus cells (NPCs), notochordal cells (NCs), annulus fibrosus cells (AFCs), end plate cells (EPCs), bone marrow‐derived mesenchymal stem cells (BM‐MSCs), nucleus pulposus‐derived mesenchymal stem cells (NP‐MSCs), human umbilical vein endothelial cell (HUVECs), disc cells (DCs), cartilaginous endplate stem cells (CESCs). Not shown: All ex vivo studies (*n* = 3) used rats IVD cultured in serum‐containing medium for EV functional studies and administered EVs via intradiscal injection.

Most of the functional studies (82 of 128) evaluated the effect of EVs in in vitro models (Figure 8A; Table S13). Limited studies used ex vivo models (*n* = 3) (Table S14), and approximately one‐third of the studies (*n* = 43) tested EV functionality in vivo models (Figure 8A; Table S15). Categories that were best reported (above 95%) across in vitro, ex vivo, and in vivo functional studies were the model species, the degeneration models, and the inclusion of a negative control, represented by control medium. Dose–response assessment and EV‐specificity controls, represented by whole conditioned medium, EV‐depleted conditioned medium, or the fraction of the conditioned medium containing soluble proteins (extracellular proteins [EP]), were scarcely reported (Tables S13–S15). These are methodological controls defined by the MISEV to assess EV‐mediated effects and allow for the evidence‐based study of the EV's regenerative potential in the IVD field. When EV‐depleted serum was used for cell or tissue culturing, the depletion protocol was rarely reported (Tables S13 and S14). In addition, categories best reported (above 95%) specifically *to* in vitro, ex vivo, or in vivo were in vitro model type, in vitro and ex vivo experimental model, ex vivo donor sex and age, explant culture medium composition, ex vivo and in vivo administered EV amount, route of administration, and treatment period. Furthermore, the information categories that were less well reported specifically for in vitro studies were the sex of the cell donor (33%) (Table S13), for ex vivo studies, the health condition of the animal sacrificed to collect explants (0%) (Table S14), and for in vivo studies, the animal health and housing conditions (44% and 30%, respectively) (Table S15).

Complete information about the specific model used in EV functional studies is necessary to interpret the reported functional outcomes and the observed biologic effects. Especially since the EV‐mediated biologic effects are context‐dependent. NP tissue presents with a well‐known diversity in cellular composition across different species [27, 28], and this context is needed when evaluating the translatability of the EV‐specific effect for an allogenic or xenogeneic treatment. Furthermore, in vitro models, whether monolayer or 3D, can have an impact on EV‐mediated effects, due to, e.g., variations in terms of EV diffusion and surface interaction with the recipient cells/tissue and different medium compositions. For ex vivo and in vivo models, the route of EV administration is a factor that impacts the final effect of the EVs, in terms of target cell and tissue interaction and EV diffusion. Moreover, EVs have been quantified in different ways, using different EV parameters and normalizations, making it impossible to compare the different EV dosages used. In addition, the critical lack of dose–response studies drastically limits the understanding and the determination of the optimal EV therapeutic dosage. All these aspects require rigorous reporting to allow for post hoc analysis and to determine the translational value of reported work.

##### 3.6.1.1 Species and Set Up of Models for EV Functional Studies

All but one of the in vitro studies reported the cell donor species of the model and studied the effects of the EVs on monolayer NPCs, NCs, annulus fibrosus cells (AFCs), and endplate cells (EPCs) (Figure 8B–D,F and Table S13). EV functional studies have been performed in different species in vitro, i.e., 56% were performed in human models, followed by rat (28%), mouse (10%), bovine (2%), and canine (2%) models, while two in vitro studies did not report the cell type used. Although all three ex vivo studies used rats, the in vivo studies entailed rats (79%), mice (19%), and rabbit (2%) (Figure 8B,F). The predominant use of rodent models ex vivo and in vivo brings translational limitations for EV applications in clinic, given the biological differences between rodents and humans IVDs [59]. Three studies tested the isolated EVs ex vivo in rat IVDs via intradiscal injection: in two studies, caudal IVD were used, while the third study did not report the spinal segments used. All in vivo studies spanning the period 2016–2023 locally administered EVs through intradiscal injection, except one study that administered EVs through intravenous injection, however, without showing EVs successfully reached the IVD (Figure 8G). Notably, xenogeneic EVs were tested in 65 in vitro studies (79%), in one ex vivo study (33%), and in 17 in vivo studies (39%). This raises potential challenges and limitations, for example, caused by differences between species in receptor–ligand interactions, as well as biological compatibility issues, which in cross‐species approaches could lead to underestimated functional EV effects.

##### 3.6.1.2 In Vitro and Ex Vivo Culture Medium Composition

Of the 82 in vitro studies, 74 reported the composition of the cell culture medium, one‐third used serum‐free medium, while the majority used serum‐containing medium (48 studies) (Figure 8E). Of these 48 studies, only 2 studies depleted the supplemented serum from exogenous EVs of which only one reported the protocol that was used to deplete EVs from the serum (Figure 8E). Moreover, in the three studies on ex vivo explants, serum‐containing medium was used, however, without reporting whether and which protocol was used to deplete the exogenous EVs from the serum.

Hereby, it cannot be fully excluded that serum‐derived EVs used to supplement the culture media may have contributed to the observed EV‐mediated effects. The MISEV guidelines recommend using serum‐free medium in EV functional studies, which avoids the aforementioned challenges and limitations of serum, such as contamination with exogenous EVs. Nonetheless, if serum supplementation is necessary, it is advised to deplete EV from the serum to prevent interference with the functional effects of the EVs being tested. Moreover, appropriate reporting of the EV‐depletion protocol is recommended to facilitate interpretation and enhance translatability of the EV studies' outcomes.

### 3.7 EV Research Progression in the IVD Field

**FIGURE 9:**
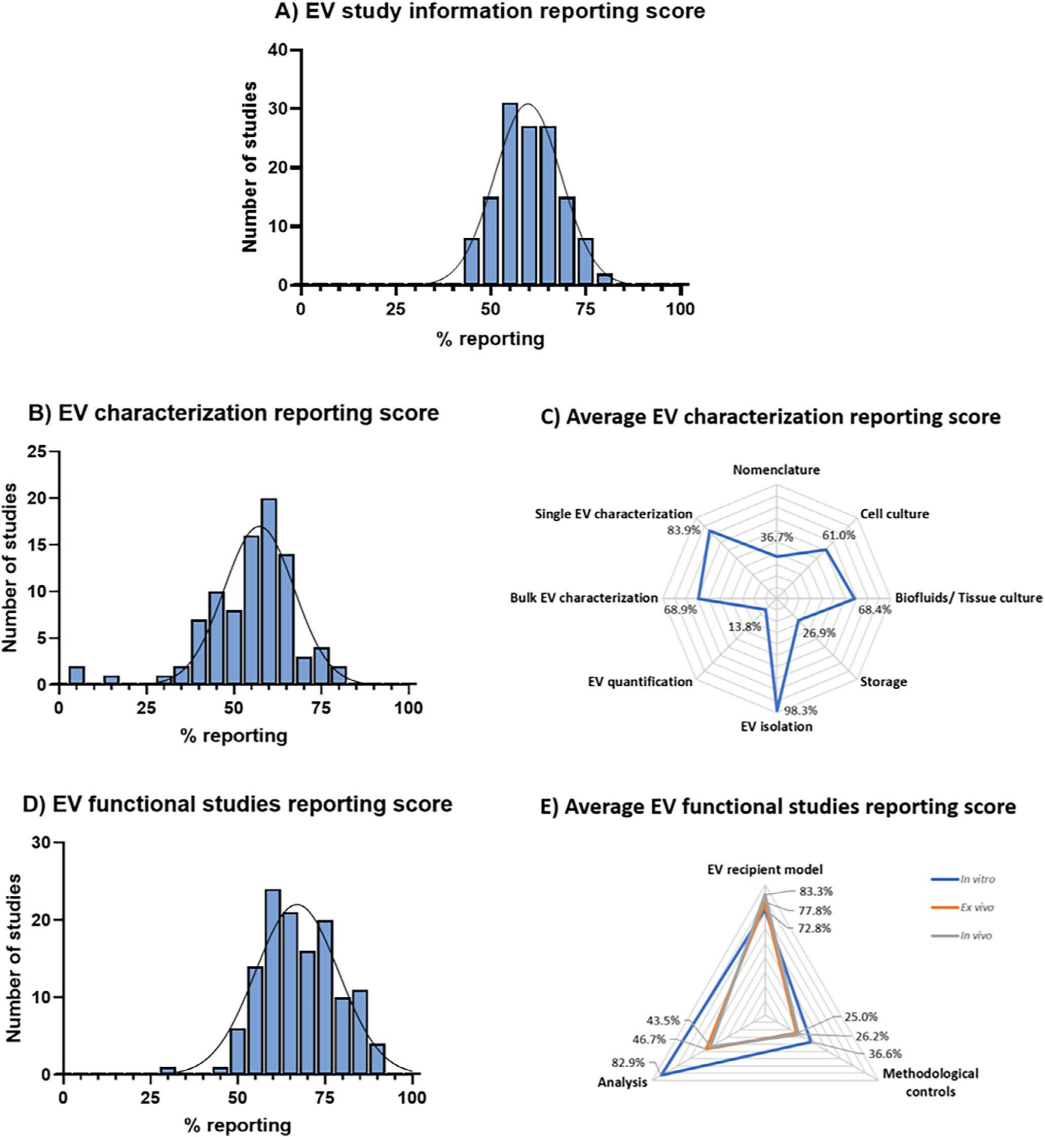
Summary of EV study information reporting rates and respective EV characterization and EV functional studies over the period 2016–2023. Histogram of the 133 studies based on their average rate (A). (B) Histogram of the study information rates and (C) average reporting percentages of the single EV characterization categories (*n* = 90 studies). (D) Histogram of the study information rates and (E) average reporting percentages of the single EV functional studies macro‐categories (*n* = 128 studies).

Among the total 133 studies included in the primary analysis spanning 2016–2023, on average, 60% of the EV study information was reported (Figure 9A). This indicates an opportunity for the IVD research field to improve reporting details of EV studies, in line with the MISEV guidelines, to better support claims of EV‐mediated effects in the IVD. Within the timespan 2016–2023, the study of Hingert et al. (2020) [60] complied the most to the MISEV guidelines and had the highest information reporting percentage (81%), representing a valuable example for the IVD field for reporting EV study information.

To better understand the different contributors to this overall reporting rate, we dissected the data into EV characterization and EV functional studies. Among the 90 EV characterization studies, on average 57% of the information was reported (Figure 9B) and Bari et al. (2018) [61] had the highest information reporting percentage (81%) over the period 2016–2023. To highlight the strengths and improvement points for the IVD field, the average reporting percentage of the macro‐categories regarding EV characterization studies (Tables S6–S12) was summarized (Figure 9C). In the 90 studies concerned, the information regarding the EV isolation was the best reported, while nomenclature, EV storage, and quantification were on average less well reported (Figure 9C).

Next, among the 128 EV functional studies, on average 67% of the information was reported (Figure 9D) with valuable examples over the period 2016–2023, such as the studies of Dai et al. (2023) [62] and Lan WR et al. (2019) [63], that on average had a 91% reporting rate. Moreover, the average reporting percentage of the respective macro‐categories (Tables S13–S15) was summarized (Figure 9E). In particular, the information regarding the overall EV recipient model and the in vitro functional study analysis was the best reported (Figure 9E). On the contrary, the inclusion of methodological controls was overall less well reported, as well as the in vivo and ex vivo functional study analysis (Figure 9E).

In summary, the aspects discussed throughout this review (and reunited in Figure 9) are essential to report to ensure scientific rigor and reproducibility of EV studies in the IVD field. However, in the IVD field, the information categories EV nomenclature, storage, EV functional studies methodological control, and analysis are less well reported and thus warrant further attention (Figure 9C,E; Table S16). The lack of inclusion of methodological controls in EV functional studies prompts questions about whether the reported outcomes are EV‐specific effects, rather than biologic effects of co‐isolates. For clinical translation of EV therapeutics in the IVD field, the inclusion of such methodological controls in functional analysis studies is of utmost importance, as well as complete and comprehensive reporting of EV study information. When it comes to clinical translation, the therapeutical potential of EV‐based therapeutics comes with challenges regarding scalability, reproducible batch preparations, and regulatory affairs [64]. Rigorous research will help in better understanding the mechanism of action of EV‐therapeutics and their potential clinical applications. Furthermore, for the IVD field, patient stratification is required to better match the most effective therapeutical approach to the specific disease staging [65]. To the authors' knowledge, there is currently no clinical study investigating the therapeutic effects of an injectable‐EV preparation to the degenerate IVD [66].

Reporting of EV study information is not only relevant for the IVD field, but also for other fields. To promote consistency across the entire EV field, the ISEV recommends the online platform EV‐TRACK (Extracellular Vesicle—Transparent Reporting and Centralizing Knowledge; www.evtrack.org [67]) as a useful knowledge platform and database designed to improve transparency, robustness, and reproducibility in EV research. Out of all the articles studied (129 articles including the primary analysis and the new analysis to cover the level of evidence), only one article was uploaded in EV‐TRACK [68]. How well the IVD field reports the relevant information regarding EV studies was put into perspective and compared to other research fields. The average EV study information reporting score derived from the present analysis (i.e., 60%) was compared with the average information reporting score derived from EV‐TRACK of the same time span (2016–2023), comprising 1973 EV studies from all research fields, which was 27%. Here, it is important to consider that EV‐TRACK categorizes and reports the EV study information differently compared to the present scoping literature review, precluding head‐to‐head comparison of the percentages. The main differences lie in the IVD‐specific information categories that are included in our scoring. Altogether, this indicates that the IVD field is performing and reporting EV studies relatively well, translating to good transparency and data interpretation. Nonetheless, there is room for improvement in better reporting to also generate robust evidence for EV‐specific therapeutic effects.

### 3.8 The EV‐idence in the IVD Field

Over the period 2016–2025, 129 peer‐reviewed articles intended to explore the EV functionality in the IVD field (Figure 2; Tables S2 and S5). In the vast majority of these studies, the effects of EV‐enriched secretome formulations were investigated. These EV‐enriched secretome formulations contain, besides EVs, also other colloidal macromolecular structures, proteins, including enzymes, cytokines, and extracellular matrix components, non–EV‐associated nucleic acids, like miRNAs and metabolites. A large body of this literature encompasses mechanistic studies, showcasing miRNAs enriched in the EV‐enriched secretome formulations and studying the underlying mode of action [69, 70, 71, 72, 73, 74, 75, 76, 77, 78, 79, 80, 81, 82, 83, 84, 85, 86, 87, 88, 89, 90, 91, 92, 93, 94, 95, 96, 97, 98, 99, 100, 101, 102, 103, 104]. In addition, studies show that EVs can be used as a drug delivery system by genetically engineering cells to overexpress target miRNAs or proteins, known to be secreted also in association with EVs, or via encapsulation [105, 106, 107, 108, 109, 110, 111, 112]. Overall, EV‐enriched secretome studies have revealed diverse biological effects involved in regenerative processes, including modulation of cell proliferation, migration, and differentiation into matrix‐producing phenotypes (Supporting Information S1). Furthermore, EV‐enriched secretome studies showed the potential of regulating various stress‐related and cell death pathways, inflammation, immune responses, angiogenesis, and endoplasmic reticulum stress, highlighting the broad role in disc homeostasis and regeneration (Supporting Information S1). A few studies have further linked secretome biologic activity to pain‐related mechanisms [106, 113, 114], suggesting the potential relevance of EVs in the pathophysiology and treatment of discogenic pain. However, in fundamental mechanistic studies intending to unequivocally demonstrate that the observed effects are EV‐mediated rather than induced by co‐isolated bioactive components, inclusion of the key methodological MISEV controls is warranted.

To determine the up‐to‐date body of “EV‐idence” for EV‐specific therapeutic effects on IVD degeneration, the literature search was updated on 1st September 2025, following the aforementioned methodology. A total of 50 new articles were added to the previously identified 79 articles, resulting in 129 peer‐reviewed articles (Figure 2; Tables S2 and S5). These 50 new articles comprised a total of 83 individual EV functional studies that were added to the previously identified 128 EV functional studies (Figure 2). Altogether, 211 EV functional studies were assessed for their inclusion of the essential MISEV methodological controls: (1) depletion of EVs from serum used in tissue or cell culture medium during EV production and EV functional studies, and (2) procedural and analysis controls, such as an EV‐depleted control or a whole conditioned medium. An EV‐depleted control derived from an EV‐enriched secretome formulation represents, hereby, the secretome depleted from EVs and can serve as an appropriate control dissecting EV‐mediated effects from the biologic effects exerted by co‐isolated biomolecules in the absence of EVs.

Out of the 211 EV functional studies analyzed, eight EV‐enriched secretome studies, derived from 5 articles, included the essential MISEV methodological controls required to substantiate an EV‐specific effect (Table S17). The reported EV‐associated regenerative effects on multiple facets of the IVD degeneration have been synthesized below (Table 2).

**TABLE 2:** Summary of the findings and results of the articles that reported all the essential information to claim an EV‐specific effect.

Article		EV functional study					
References	EV source	Set up	Model species	EV recipient	Stress stimuli	EV treatment	Results
Lan (2019) [63]	SD rat NCs	In vitro	SD rat	BM‐MSCs	—	EVs for 7, 14, and 21 days	Increase Col2, Acan and Sox9 gene and protein expression, and Cd24 and Krt19 protein expression Decrease Notch1, Hes1 and Hey1 gene and protein expression Dose‐dependent increase of MSCs migration
González‐Cubero (2022) [115]	Human Ad‐MSCs	In vitro	Human	AFCs	TNF	Stimulus + EVs for 12 h	Increase MMP‐3 and MMP‐13 protein expression Counteract *IL1B, IL6*, and *IL17* gene expression Increase *NGF* and *BDNF* gene expression
			Human	NPCs	TNF	Stimulus + EVs for 12 h	Preserve MMP‐3 protein expression Counteract *TNF and IL6* gene expression Increase *BDNF* gene expression
Qian (2022) [116]	SD rat PRP	In vivo	SD rat	Co5/6 IVD	Needle puncture	Stimulus + EVs for 4 weeks	Counteract Casp‐1‐immunopositive cells Counteract Il1β, Casp‐1, Nlrp3, Bax, and cleaved Casp‐3 protein expression Preserve Bcl2 protein expression
Dai (2023) [62]	Human PRP	In vitro	SD rat	NCs	H_2_O_2_	Stimulus + EVs for 12 h	Counteract ROS+ cells, apoptotic (PI/FITC‐A+ and TUNEL+) cells and senescent (SA‐b‐GAL+) cell numbers Counteract *Cdkn2a* and *Cdkn1a* gene expression Preserve Col2, Sox9, and Acan gene and protein expression Counteract Mmp‐3, Mmp‐13, and Adamts‐5 gene and protein expression Preserve Col2 and Acan cell immunopositivity Counteract Mmp‐13 and Adamts‐5 cell immunopositivity Preserve Ppargc1a cell immunopositivity Preserve Sirt1, Ppargc1a, Tfam, Nrf1 and Nrf2 gene and protein expression Counteract mitochondrial ROS (MitoSOX‐red cell positivity) Recover mitochondrial functionality + (Mitotracker green cell positivity) Preserve mitochondrial oxidative stress in early apoptosis (restore JC‐1 monomers cell positivity and recover JC‐1 aggregates cell immunopositivity) Counteract lactic acid levels Preserve ATP levels
		In vivo	SD rat	Co7/8 IVD	Needle puncture	Stimulus for 1 week + EVs for 4 and 8 weeks	Preserve water content (T2‐weighted MRI signal) over time Preserve disc height (DHI) over time Improve histological degeneration grade Slightly maintain Col2 cell immunopositivity Reduce Mmp‐13 cell immunopositivity Reduce ROS levels (DHE cell immunopositivity) Reduce mitochondrial ROS level (MitoSOX‐red cell positivity) Restore Pgc1a cell immunopositivity to healthy levels
van Maanen et al. (2025) [117]	Porcine NC‐rich NP explant	In vitro	Canine	3D NPCs	IL1β	Stimulus + EVs for 1 week	Decrease GAG release
		Ex vivo	Human	NP explant	IL1β	Stimulus + EVs for 2 weeks	Decrease IL6 release

#### 3.8.1 EV‐Mediated Modulation of MSC Migration and Differentiation in Vitro

Lan et al. shows that EVs originating from expanded 8‐week‐old rat NCs promoted the upregulation of NPC markers, such as Sox9, Cd24, and Krt19, and downregulation of Notch1, Hes1, and Hey1 protein expression in rat bone marrow–derived MSCs (BM‐MSCs) [63]. In addition, EVs derived from cultured rat NCs increased collagen 2a1 and aggrecan at the gene and protein levels in rat BM‐MSCs in vitro [63], indicating differentiation of BM‐MSCs toward a chondrogenic‐producing cell phenotype. Moreover, these cultured rat NC‐derived EVs promoted BM‐MSCs migration in a dose‐dependent manner in vitro, which is considered by Lan et al. a promising strategy for replenishment of NPCs in the attempt of IVD regeneration [63]. In light of the MISEV guidelines, some caveats were identified. The EV‐depletion protocol, both for serum‐EV depletion and EV depletion from conditioned medium as methodological control, was much shorter (120 000 g for 90 and 70 min) [63] than recommended by the ISEV (> 100 000 g for > 18 h) [16]. Consequently, the serum supplemented in the culture media during EV production, as well as the EV‐depleted media used as methodological control, could still contain serum‐derived EVs. Moreover, IVDs of 8‐week‐old rats are rich in NCs, which are known to lose their vacuolated phenotype upon culture in monolayers and in the presence of FBS [118]. Therefore, the tested EVs reported as NC‐derived may originate from cells with a different phenotype. In conclusion, these EVs enhanced MSC migration, but further characterization of the EVs is necessary to confirm their cellular origin.

#### 3.8.2 EV‐Mediated Extracellular Matrix Metabolism of NPCs In Vitro

One study investigated the EV‐specific effects at the ECM level in a canine NPC pellet culture and a human NP explant model [117], using EVs derived from porcine NC‐rich NP tissue and retrieving inconclusive findings. One‐week treatment of NC‐EVs attenuated GAG release from canine NPC pellets challenged with 1 ng/mL IL1β. However, this protective effect was not confirmed after 2 weeks of NC‐EV media supplementation in human NP explants stimulated with 0.1 ng/mL IL1β. Noteworthy, the authors evidenced the importance of determining the background levels of EV‐enriched and EV‐depleted treatment controls. In the specific study, conditioned media were derived from ECM‐rich tissues, like the NC‐rich NP tissue of healthy pigs. Likewise, proteomic analysis of the EV‐associated protein cargo derived from human NP tissue from different degeneration stages by Li et al. confirmed the presence of ECM molecules associated and possibly also co‐isolated with EVs [31]. It is well known that the ECM signals through its molecules, as well as through the growth factors and cytokines harbored within the ECM. Altogether, this implies that when testing EV‐enriched formulations derived from tissues that are rich in their ECM, EV‐specific effects should be demonstrated experimentally with the use of appropriate EV‐depleted methodological controls. Furthermore, additional considerations involve studies using EVs derived from disc tissues with variable degeneration levels, which inherently represent tissues with differential cellular and matrix composition. In this context, Li et al. demonstrated that mildly degenerated human NPCs released more EVs compared to non‐degenerated and degenerated human NPCs. Moreover, the protein cargo composition reflected the degeneration grade of the EV donor: EVs derived from non‐degenerated NPCs were enriched with proteins involved in cell adhesion and ECM–receptor interaction; those from mildly degenerated NPCs‐derived EVs showed enrichment for ECM organization and structure; and EVs from degenerated NPCs‐derived EVs were enriched in proteins linked to vesicle‐mediated transport [31]. While the observed dynamics in the EV protein cargo can serve as a biomarker providing insights into tissue homeostasis, it also unveils that EV‐enriched secretome studies might require normalization among treatment groups in a study design. Dependent on the research question, study designs for EV‐specific effects may furthermore require either normalization for the number of vesicles added or normalization based on the mL media/tissue weight used to generate EVs from tissues or mL media/number of cells used in culture.

#### 3.8.3 EV‐Mediated Modulation of Inflammatory and Oxidative Stress Responses of NPCs, AFCs, and NCs in Vitro

To date, three studies investigated the protective EV‐specific effects of EVs during inflammatory or oxidative stress on NPCs in vitro [62, 115] and ex vivo [117], using proinflammatory stimuli (i.e., TNF or IL1β), or an oxidative‐stress inducing stimulus (H_2_O_2_). González‐Cubero et al. showed that EVs derived from human adipose tissue‐derived MSCs counteracted TNF‐induced (25 ng/mL) pro‐inflammatory cytokine expression (*IL1B, IL6*, and *IL17*) in human AFCs, while the expression of *TNF*, *IL1A*, *CXCL8*, and *IFNG* was not modulated specifically by EVs [115]. Furthermore, in human NPCs, these EVs counteracted TNF‐induced *TNF* and *IL6* expression, while no EV‐specific effect was observed for other cytokines (*IL1A*, *IL1B*, *CXCL8*, *IL17*, and *IFNG*). These findings were confirmed by demonstrating that also translocation of NF‐κB to the nucleus, a marker of NF‐κB activation, was reduced in NPCs in the presence of EVs as opposed to the soluble fraction controls. It is unclear whether these protective effects translate into beneficial downstream modulation of the TNF‐induced inflammation effects at the ECM level. In the presence of TNF, taking into consideration the methodological controls of conditioned media and the soluble fraction, human MSC‐derived EVs increased MMP release by human NPCs (MMP‐3) and by human NPCs and AFCs (MMP‐13), while no EV‐specific effect was observed for MMP‐1, MMP−2, and ADAMTS‐5. Consistent with these findings, human MSC‐derived EVs increased the expression of the neurotrophin *BDNF* both in human NPCs and AFCs compared to the conditioned medium and soluble fraction, as well as *NGF* gene expression in human AFCs upon TNF stimulation [115]. This study indicates that MSC‐EVs could modulate NF‐κB signaling and matrix remodeling and gene expression of cytokines and neurotrophic factors in NPCs and AFCs; however, the cumulative effects at the tissue level require further studies. Noteworthy, this study used an EV‐depletion protocol for EV media as a methodological control (100 000 g for 70 min twice) [115] shorter than recommended by the ISEV (> 100 000 g for > 18 h) [16]. Nonetheless, this study took along whole conditioned medium, which is an accepted methodological control by the ISEV in EV functional studies.

van Maanen et al. treated canine NPC pellets with EVs derived from porcine NC‐rich NP tissue and 1 ng/mL IL1β for 1 week [117], and reported reduced IL6 release compared to IL1β controls. However, this effect was also observed in the EV‐depleted control condition, indicating a non‐EV‐mediated decrease in IL6 release. Notably, EV‐depleted media increased PGE_2_ release from NPC pellets, with and without IL1β stimulation, while PGE2 release was unaffected by the EV treatment. Altogether, this indicates that the observed anti‐inflammatory effects were not specifically mediated by NC‐EVs. Contrary to these findings, NC‐EVs reduced IL6 and CXCL1 release from human NP explants stimulated with 0.1 ng/mL IL1β [117], whereas this effect was lost under EV‐depleted conditions. Moreover, human NP explants treated with NC‐EVs alone for 2 weeks presented an increased CCL2 release, while CCL2 was unaffected in the EV‐depleted condition. These results indicate that porcine NC‐EVs can mediate immunomodulatory effects on human NPCs in short‐term tissue culture.

To study the rescue potential of EVs during oxidative stress stimuli in vitro, Dai et al. tested human PRP‐derived EVs on rat cultured NCs exposed to H_2_O_2_ (200 μM), finding that EVs, compared to whole PRP, partially rescued cultured rat NCs from apoptosis, senescence, and oxidative stress, with slight reduction of the cell cycle inhibitors *Cdkn2a* and *Cdkn1a*, as well as improving expression of mitochondrial proteins and mitochondrial function [62]. These effects translated also to beneficial effects at the ECM level. PRP‐derived EVs preserved Col2 and Acan gene and protein expression. They also counteracted Mmp‐3, ‐13, and Adamts‐5 gene and protein expression [62]. This suggests that PRP‐derived EVs could improve cell survival and matrix production of cultured rat NCs that are challenged by oxidative stress. These findings require replication in human NPCs to determine the translational potential of PRP‐derived EVs.

#### 3.8.4 PRP EV‐Mediated Improvement of Degenerated IVD In Vivo

Two studies tested the disease‐modifying properties of EVs on an in vivo rat IVD degeneration model [62, 116]. Both studies induced degeneration of the IVD via needle puncture. Qian et al. intradiscally injected rat PRP‐derived EVs immediately after the induction, while Dai et al. intradiscally injected human PRP‐derived EVs 1 week after the induction of IVD degeneration. Both studies included whole PRP as a methodological control for their EV functional study (Table S17). In line with the above‐described in vitro results, rat PRP‐derived EVs counteracted the release of IL1β and cell apoptosis when administered directly after the induction of IVD degeneration [116]. This was demonstrated by reduced numbers of Caspase‐1‐positive cells, and reduced levels of pro‐apoptotic factors (Casp‐1, Bax, and cleaved Casp‐3 production), and increased levels of the anti‐apoptotic factor Bcl2 [116].

Furthermore, human PRP‐derived EVs maintained Col2‐positive and reduced Mmp‐13‐positive cells in rat IVDs, where treatment was administered 1 week after the induction of IVD degeneration by needle puncture in vivo [62]. Furthermore, the percentage of ROS‐positive cells and PGC1α (a major regulator of mitochondrial biogenesis) was comparable to healthy IVDs [62]. These results indicate the ability of PRP‐derived EVs to regulate cellular stress in the early phase of induced IVD degeneration.

At the tissue level, PRP‐derived EVs have been reported to be beneficial in mitigating IVD degeneration [62, 116]. Rat PRP‐derived EVs, intradiscally administered directly after induction of IVD degeneration, were claimed to preserve the water content within the IVD, counteract histological AF ring distortion, AF‐NP boundary blurring, NP matrix density, and NP cellularity loss. However, these findings were based on descriptive data, without further support from quantifications at the level of the T2 intensities of the NP on MRI, nor histological scoring [116]. Similarly, human PRP‐derived EVs, intradiscally delivered one week after induction of IVD degeneration, preserved the water content within the IVD and disc height over time, both evaluated quantitatively, and halted the development of IVD degeneration at the histological level [62].

While these results pave the way for a better understanding of the PRP‐derived EV‐specific role and effect within the IVD and its degeneration process, the reported studies differ considerably in the EV source, the experimental setup, and the models used to test the EV‐specific effect. Even more so, the majority of the in vitro and all in vivo evidence provided uses the rat as a model. Rats commonly contain NCs in healthy discs and present with a much better regenerative potential than human discs, which are devoid of NCs and scarcely populated with the smaller non‐vacuolated NP cells. Hence, it remains elusive whether PRP‐derived EVs will demonstrate similar regenerative capacity within the degenerate human IVD disc.

## 4 Conclusions and Implications for Clinical Translation

In this scoping literature review, we discuss the methodological MISEV requirements and provide considerations for functional studies exploring EV‐specific effects within the IVD field. Analysis of the current literature revealed that the quality of reporting IVD‐related methodological information is above average compared to the general EV field. Furthermore, fundamental and translational studies concerning EV‐enriched formulations exploring therapeutic approaches and showcasing beneficial effects in a broad range of processes involved in IVD regeneration are expanding. To date, there are however limited studies that provide fundamental mechanistic insights in EV‐mediated modulation of regenerative processes, substantiated with the MISEV methodological controls (i.e., the use and full reporting of the generation of EV‐depleted serum used to supplement culture media, and the use of EV‐depleted controls or conditioned media in functional analysis studies). However, for clinical translation, EV‐enriched secretome studies provide valuable perspectives, and GMP‐compatible production of EV therapeutics is possible [119]. Co‐isolates present in such EV therapeutics may contribute to the observed biologic effects, either as part of the biomolecular corona of EVs or by acting in concert with EV‐mediated effects [120]. Importantly, the co‐isolates will vary between different EV‐enrichment strategies. In this context, a standardized methodology for EV enrichment or isolation is an illusion since the process defines the product. Of course, the selected process should be robust and reproducible, and CQAs (critical quality attributes) of the EV‐therapeutics should be defined for each EV‐based therapeutic. This review strives to create awareness of the fact that each enriched EV preparation has features that are affected by the isolation method and that can influence the functionality. To conclude, for the observed effect of an EV‐enriched preparation, EVs are essential; proper methodological and procedural controls need to be included. Altogether, this indicates that with the right methodology and reporting, there is a great chance for the emerging and fast‐expanding EV field to unlock the therapeutical potential of EVs for IVD degeneration.

## Author Contributions

**Daniele Corraini:** writing – original draft, writing – review and editing, visualization, methodology, investigation, formal analysis, data curation. **Chantal Voskamp:** writing – review and editing, validation, supervision, project administration, data curation. **Marca H. M. Wauben:** writing – review and editing, validation, supervision. **Marianna A. Tryfonidou:** writing – conceptualization, review and editing, validation, supervision, project administration, funding acquisition.

## Funding

This work was supported by Nederlandse Organisatie voor Wetenschappelijk Onderzoek, 19251. Horizon 2020 Framework Programme, 825925.

## Conflicts of Interest

The authors declare no conflicts of interest.

## Supporting information


**Data S1:** Supporting Information 1

**Data S2:** Supporting Information 2

## Data Availability

The data that support the findings of this study are available from the corresponding author upon request.
