# Supplementary material for: Regenerative Potential of Extracellular Vesicles on Intervertebral Disc Degeneration: What is the EV‐idence?
Source: JOR Spine. 2025 Dec 25;8(4):e70149. doi: 10.1002/jsp2.70149 (PMC12740142; doi:10.1002/jsp2.70149)
Supplement: Supplementary file 2 — Data S2: Supporting Information 2 [file JSP2-8-e70149-s001.docx]

**Supplementary Files of “Regenerative potential of extracellular vesicles on intervertebral disc degeneration: What is the EV-idence?”**

**Table of Contents**

[**PubMed Query -** The search query used to identify IVD-related EV research articles: 2](#_Toc212725423)

[**Supplementary table 1** – List of 14 articles (published between 1988 – 2023) excluded from the MISEV guidelines compliance analysis (*sections 2 – 7*) and the EV-idence analysis (*section 8*). 2](#_Toc212725424)

[**Supplementary table 2** – List of 79 articles (published between 2016 – 2023) included in the MISEV guidelines compliance analysis (*sections 2 – 7*) and the EV-idence analysis (*section 8*). 2](#_Toc212725425)

[**Supplementary table 3** – Comparison of information categories for EV studies between MISEV 2018 guidelines and the present scoping review. 5](#_Toc212725426)

[**Supplementary table 4** – List of 3 new articles (published between 2024-2025) excluded from the EV-idence analysis (*section 8*). 11](#_Toc212725427)

[**Supplementary table 5** – List of 50 new articles (published between 2024-2025) included in the EV-idence analysis (*section 8*). 11](#_Toc212725428)

[**Supplementary table 6** – Summary table of the average reporting percentages regarding the information on EV production from cell culture**.** 13](#_Toc212725429)

[**Supplementary table 7** - Summary table of the average reporting percentages regarding the information on EV production from biofluids or tissue culture. 14](#_Toc212725430)

[**Supplementary table 8** - Summary table of the average reporting percentages of storage of biofluids, conditioned medium or isolated EVs. 15](#_Toc212725431)

[**Supplementary table 9** - Summary table of the reporting percentages regarding the information on isolation and concentration methods used to enrich for EVs. 15](#_Toc212725432)

[**Supplementary table 10** – Frequency of each method used as primary technique or in combination with a sequential method to further enrich for EVs. 15](#_Toc212725433)

[**Supplementary table 11** - Summary table of the reporting percentages for quantification methodology of isolated EVs. 16](#_Toc212725434)

[**Supplementary table 12** - Summary table of the reporting percentages regarding characterization of isolated EVs. 16](#_Toc212725435)

[**Supplementary table 13** - Summary table of the average reporting percentages for EVs functional studies *in vitro*. 17](#_Toc212725436)

[**Supplementary table 14** - Summary table of the average reporting percentages for EVs functional studies *ex vivo*. 18](#_Toc212725437)

[**Supplementary table 15** - Summary table of the average reporting percentages for EVs functional studies *in vivo*. 19](#_Toc212725438)

[**Supplementary table 16** – Summary table of the average information reporting percentages of the 133 EV studies per category. 20](#_Toc212725439)

[**Supplementary table 17** – Summary table of the studies reporting the minimal essential information (EV-depletion serum protocol) and including the methodological controls (EV-depleted medium or conditioned medium). 21](#_Toc212725440)

### **PubMed Query -** The search query used to identify IVD-related EV research articles:

*("intervertebral disc" AND "extracellular vesicles") OR ("endplate" AND "extracellular vesicles") OR ("nucleus pulposus" AND "extracellular vesicles") OR ("annulus fibrosus" AND "extracellular vesicles") OR ("intervertebral disc" AND "exosomes") OR ("endplate" AND "exosomes") OR ("nucleus pulposus" AND "exosomes") OR ("annulus fibrosus" AND "exosomes") OR ("intervertebral disc" AND "microvesicles") OR ("endplate" AND "microvesicles") OR ("nucleus pulposus" AND "microvesicles") OR ("annulus fibrosus" AND "microvesicles") NOT (Review[Publication Type]) NOT (Meta-analysis[Publication Type]) NOT (Systematic Review[Publication Type]) NOT (Preprint[Publication Type]) NOT (Retracted Publication[Publication Type]) NOT (Retraction of Publication[Publication Type]) NOT (Abstract[Publication Type]) NOT (Scientific Integrity Review[Publication Type]) NOT (Comment[Publication Type]) NOT (Guideline[Publication Type]) NOT (English Abstract[Publication Type]) NOT (Published Erratum[Publication Type])*.

### **Supplementary table 1** – List of 14 articles (published between 1988 – 2023) excluded from the MISEV guidelines compliance analysis (*sections 2 – 7*) and the EV-idence analysis (*section 8*).

| **PMID** | **First Author** | **Journal** | **Publication Year** |
| --- | --- | --- | --- |
| 2969690 | Kubota M | Anat Anz | 1988 |
| 2182650 | Baumert M | J Cell Biol | 1990 |
| 22438989 | Strassburg S | PLoS One | 2012 |
| 34000376 | Shi M | Spine J | 2021 |
| 34966192 | Kraus P | Biocell | 2021 |
| 34636308 | Zhang XB | Curr Gene Ther | 2022 |
| 35720640 | Morteza Bagi H | Neurobiol Pain | 2022 |
| 36370754 | Wang Y | Biochem Pharmacol | 2022 |
| 36429018 | Li L | Cells | 2022 |
| 35450531 | Hechavarria ME | Curr Stem Cell Res Ther | 2023 |
| 37008028 | Tilotta V | Front Bioeng Biotechnol | 2023 |
| 37122729 | Ren H | Front Immunol | 2023 |
| 37150986 | Wang H | Curr Stem Cell Res Ther | 2023 |
| 37930732 | Li QW | Tissue Eng Part C Methods | 2023 |

### **Supplementary table 2** – List of 79 articles (published between 2016 – 2023) included in the MISEV guidelines compliance analysis (*sections 2 – 7*) and the EV-idence analysis (*section 8*).

| **PMID** | **First Author** | **Journal** | **Publication Year** |
| --- | --- | --- | --- |
| 27572543 | Bach FC | Eur Cell Mater | 2016 |
| 28460630 | Moen A | J Transl Med | 2017 |
| 28486958 | Lu K | Stem Cell Res Ther | 2017 |
| 29179481 | Bach F | Oncotarget | 2017 |
| 28805297 | Cheng X | J Cell Mol Med | 2018 |
| 30380806 | Bari E | Cells | 2018 |
| 30187277 | Qi L | J Bone Miner Metab | 2019 |
| 30892812 | Yuan FL | J Cell Mol Med | 2019 |
| 31249601 | Lan WR | Stem Cells Int | 2019 |
| 31281533 | Liao Z | Theranostics | 2019 |
| 31351174 | Xia C | Free Radic Biol Med | 2019 |
| 32436493 | Li M | Med Sci Monit | 2020 |
| 32464126 | Zhu G | Exp Cell Res | 2020 |
| 32620067 | Yuan Q | Stem Cells Dev | 2020 |
| 32635856 | Zhu L | Cell Cycle | 2020 |
| 32727623 | Hingert D | Stem Cell Res Ther | 2020 |
| 32771218 | Li ZQ | Am J Med Sci | 2020 |
| 32828826 | Hu SQ | Exp Cell Res | 2020 |
| 32858458 | Song J | Mol Ther Nucleic Acids | 2020 |
| 32860495 | Zhang J | J Cell Mol Med | 2020 |
| 33230460 | Xie L | Mol Ther Nucleic Acids | 2020 |
| 33294295 | Sun Z | Mol Ther Nucleic Acids | 2020 |
| 33488928 | Xiang H | Oxid Med Cell Longev | 2020 |
| 33038032 | Zhang QC | J Orthop Res | 2021 |
| 33217442 | Sun Z | Life Sci | 2021 |
| 33314719 | Chen CC | Orthop Surg | 2021 |
| 33459443 | Luo L | Stem Cells | 2021 |
| 33465243 | Tang S | Eur Cell Mater | 2021 |
| 33499725 | Wen T | Cell Cycle | 2021 |
| 33748142 | Luo L | Front Cell Dev Biol | 2021 |
| 33984637 | Liao Z | Biomaterials | 2021 |
| 33985571 | Sun Y | Stem Cell Res Ther | 2021 |
| 34136064 | Guo Z | Oxid Med Cell Longev | 2021 |
| 34229586 | Xu J | Cell Cycle | 2021 |
| 34252537 | Zhang Q | Cell Signal | 2021 |
| 34377430 | Cui S | J Tissue Eng | 2021 |
| 34412584 | Yuan X | Mol Med | 2021 |
| 34434488 | Sun Y | Oxid Med Cell Longev | 2021 |
| 34476937 | Liao Z | ACS Nano | 2021 |
| 34488795 | Xing H | J Nanobiotechnology | 2021 |
| 34707712 | Zhang Z | Exp Ther Med | 2021 |
| 34733401 | Hu Y | Oxid Med Cell Longev | 2021 |
| 34733928 | Zhang QC | Ann Transl Med | 2021 |
| 34777000 | Wang H | Front Physiol | 2021 |
| 35386360 | Luo L | Bioact Mater | 2021 |
| 34879291 | Zhou ZM | Acta Biomater | 2022 |
| 35111808 | Zhuang Y | Front Mol Biosci | 2022 |
| 35121152 | González-Cubero E | Spine J | 2022 |
| 35127996 | Sun Z | Regen Ther | 2022 |
| 35231441 | Chen D | Exp Cell Res | 2022 |
| 35359595 | Feng X | Front Mol Biosci | 2022 |
| 35753124 | Qian J | Int Immunopharmacol | 2022 |
| 35762224 | Hu SQ | Cell Biol Int | 2022 |
| 35814268 | Yu X | Oxid Med Cell Longev | 2022 |
| 35847582 | Yu XJ | Oxid Med Cell Longev | 2022 |
| 35855812 | Xiao Q | Stem Cells Int | 2022 |
| 35984761 | Hu B | Shock | 2022 |
| 36040157 | DiStefano TJ | Cartilage | 2022 |
| 36041665 | Hao Y | Osteoarthritis Cartilage | 2022 |
| 36123708 | Liao Z | J Nanobiotechnology | 2022 |
| 36311041 | Wang B | Stem Cells Int | 2022 |
| 36334792 | Li W | Exp Cell Res | 2022 |
| 36536658 | Dai Z | Mater Today Bio | 2022 |
| 36730125 | Tong B | ACS Nano | 2023 |
| 36731695 | Guan M | Int J Biol Macromol | 2023 |
| 36967623 | Yang B | Folia Morphol (Warsz) | 2023 |
| 37108824 | Yu Y | Int J Mol Sci | 2023 |
| 37139781 | Ekram S | Cartilage | 2023 |
| 37165721 | Peng Y | Small | 2023 |
| 37206570 | Chen XW | Exp Ther Med | 2023 |
| 37432866 | Liu C | ACS Nano | 2023 |
| 37528254 | Xu G | Stem Cell Rev Rep | 2023 |
| 37587825 | Chen F | Curr Mol Med | 2023 |
| 37667246 | Zhao X | J Nanobiotechnology | 2023 |
| 37870186 | Hu H | Adv Sci (Weinh) | 2023 |
| 37872583 | Yang L | J Orthop Surg Res | 2023 |
| 37964606 | Duan Y | Acta Biochim Biophys Sin (Shanghai) | 2023 |
| 38023721 | Liu Y | Bioeng Transl Med | 2023 |
| 38037469 | Liao Z | Clin Transl Med | 2023 |

### **Supplementary table 3** – Comparison of information categories for EV studies between MISEV 2018 guidelines and the present scoping review.

| **MISEV 2018** | | | **Scoping review: EV-idence** | | | |
| --- | --- | --- | --- | --- | --- | --- |
| **1-Nomenclature** | | | | | | |
| Generic term extracellular vesicle (EV): With demonstration of extracellular (no intact cells) and vesicular nature per this characterization (Section 4) and function (Section 5) guidelines | | | (EVs, exosomes, microvesicles, apoptotic bodies) | | | |
| Generic term, e.g., extracellular particle (EP): no intact cells but MISEV guidelines not satisfied | | |  |  |  |  |
| Generic term extracellular vesicle (EV) + specification (size, density, other) + Specific term for subcellular origin: e.g., ectosome, microparticle, microvesicles (from plasma membrane), exosome (from endosomes), with demonstration of the subcellular origin | | |  |  |  |  |
| Other specific term: with definition of specific criteria | | |  |  |  |  |
| **2-Collection and pre-processing** | | | | | | |
| **Tissue Culture Conditioned medium (CCM, Section 2-a) - cell culture** | | | | | | |
| General cell characterization (identity, passage, mycoplasma check - cell line…) | | | | Donor species (incl. strain for rodents; breed for large animals) | | |
|  |  |  |  | Donor sex | | |
|  |  |  |  | Donor age | | |
|  |  |  |  | Donor cell type | | |
|  |  |  |  | Donor health status (e.g. macroscopical, histology, MRI) | | |
|  |  |  |  | Cell culture passage | | |
|  |  |  |  | Mycoplasma test (cell line) | | |
| Medium used before and during collection (additives, serum, other) | | | | Culture medium composition (additives, serum, other) | | |
| Nature and size of culture vessels, and volume of medium during conditioning NOTE: cell/cm^2^ | | | | Culture medium volume (cells/mL) | | |
| Number of cells/ml or /surface area and % of live/ dead cells at time of collection (or at time of seeding with estimation at time of collection) | | | | Cell density (cells/cm^2^) | | |
|  |  |  |  | Live/dead cells (%) | | |
| Frequency and interval of CM harvest + culture period | | | | Culture period | | |
|  |  |  |  | CM harvest frequency | | |
| exact protocol for depletion of EVs/EPs from additives in collection medium (FBS, plasma, naturally derived matrix, purified biological additives) | | | | Medium contaminants depletion protocol (FBS, plasma, natural matrix) | | |
| specific culture conditions (treatment, % O2, coating, polarization) before and during collection/ spinner flask/ matrix | | | | Cell treatment (stimuli) | | |
|  |  |  |  | Cell culture set up (monolayer, 3D, suspension) | | |
|  |  |  |  | Culture environment (% O2, % CO2, Temperature) | | |
| **Biofluids or Tissue culture (Sections 2-b and -c)** | | | | | | |
| Donor status if available (species, age, sex, food/water intake, collection time, disease, medication, other) + biofluid/tissue type | | | | Donor species (incl. strain for rodents; breed for large animals) | | |
|  |  |  |  | Donor sex | | |
|  |  |  |  | Donor age | | |
|  |  |  |  | Sample type (tissue, biofluid) | | |
|  |  |  |  | Donor health status (e.g. macroscopical, histology, MRI) | | |
| For cultured tissue explants: volume, nature of medium and time of culture before collecting conditioned medium + culture condition + culture environment | | | | Culture medium composition (additives, serum, other) | | |
| Volume of biofluid or volume/mass of tissue sample collected per donor | | | | Collected CM (mL/gr) or biofluid volume | | |
| All known collection conditions, including additives, at time of collection | | | | Collection site (sampling location) | | |
| Pre-treatment to separate major fluid-specific contaminants before EV isolation | | | | Medium contaminants depletion protocol (FBS, plasma, natural matrix) | | |
| Temperature and time of biofluid/tissue handling before and during pre-treatment | | | | Time and temperature of biofluid or tissue collection (fresh, cadaveric) | | |
| Total volume/mass used for EV isolation (if pooled from several donors) | | | | Total pooled sample volume or tissue mass | | |
| For direct tissue EV extraction: treatment of tissue to release vesicles without disrupting cells (time, temperature, method, live/dead) | | | | Live/dead cells (%) | | |
|  |  |  |  | Culture period | | |
|  |  |  |  | CM or biofluid harvest frequency | | |
|  |  |  |  | Sample treatment (e.g. stress stimuli) | | |
|  |  |  |  | Tissue culture set up (free swelling, bioreactor, spinner flask) | | |
|  |  |  |  | Culture environment (% O2, % CO2, Temperature) | | |
|  |  |  |  | Tissue treatment method for direct EVs isolation (enzymatic activity or mg/mL, chemical-physical) | | |
|  |  |  |  | Treatment time | | |
|  |  |  |  | Treatment temperature | | |
| **Storage and recovery (Section 2-d): Biofluids, CM and EV storage** | | | | | | |
| Storage and recovery (e.g., thawing) of CCM, biofluid, or tissue before EV isolation (storage temperature, vessel, time; method of thawing or other sample preparation) | | | | CM or biofluid or tissue storage temperature | | |
|  |  |  |  | Storage vessel (low-binding tubes) | | |
| Storage and recovery of EVs after isolation (temperature, vessel, time, additive(s), method) | | | | EVs storage method (e.g. frozen, lyophilized, sucrose) | | |
|  |  |  |  | EV storage vessel (low-binding tubes) | | |
|  |  |  |  | EVs storage temperature | | |
| **3-EV isolation and concentration** | | | | | | |
| **Experimental details of the method** | | | | | | |
| Centrifugation: reference number of tube(s), rotor(s), adjusted k factor(s) of each centrifugation step (= time+ speed+ rotor, volume/density of centrifugation conditions), temperature, brake settings - INTERMEDIATE RECOVERY INTERMEDIATE SPECIFICITY | | | | EV isolation method (ultracentrifugation, density gradient, chromatography, precipitation, filtration, antibody-affinity) | | |
| Density gradient: nature of matrix, method of generating gradient, reference (and size) of tubes, bottom up (sample at bottom, high density) or top-bottom (sample on top, low density), centrifugation speed and time (with brake specified), method and volume of fraction recovery - LOW RECOVERY HIGH SPECIFICITY | | | |  |  |  |
| Chromatography: matrix (nature, pore size…), loaded sample volume, fraction volume, number - INTERMEDIATE RECOVERY INTERMEDIATE SPECIFICITY | | | |  |  |  |
| Precipitation: reference of polymer, ratio vol/vol or weight/vol polymer/fluid, time/temperature of incubation, time/speed/temperature of centrifugation - HIGH RECOVERY LOW SPECIFICITY | | | |  |  |  |
| Filtration: reference of filter type (=nature of membrane, pore size…), time and speed of centrifugation, volume before/after (in case of concentration) - INTERMEDIATE RECOVERY INTERMEDIATE SPECIFICITY | | | |  |  |  |
| Antibody-based: reference of antibodies, mass Ab/ amount of EVs, nature of Ab carrier (bead, surface) and amount of Ab/carrier surface - LOW RECOVERY HIGH SPECIFICITY | | | |  |  |  |
| Other…: all necessary details to allow replication | | | | Detailed EV isolation protocol | | |
| Additional step(s) to concentrate, if any (concentrating tube, time, speed) | | | | Concentration method (e.g. centrifugal filter tube, TFF) | | |
| Additional step(s) to wash matrix and/or sample, if any (method, time…) | | | | Matrix or sample washing method | | |
| **Specify category of the chosen EV separation/concentration method (Table 1):** | | | | | | |
| High recovery, low specificity = mixed EVs and non- EV components OR + Intermediate recovery, intermediate specificity = mixed EVs with limited non-EV components OR + Low recovery, high specificity = subtype(s) of EVs with as little non-EV as possible OR + High recovery, high specificity = subtype(s) of EVs with as little non-EV as possible | | | | EVs category (high recovery/high purity, low recovery/high purity, high recovery/low purity, low recovery/low purity) | | |
| **4-EV characterization** | | | | | | |
| **EV Quantification (Table 2a, Section 4-a)** | | | | | | |
| Volume of fluid, and/or cell number, and/or tissue mass used to isolate EVs | | | | Fluid volume or cell number or tissue mass used to isolate EVs | | |
| Global quantification by at least 2 methods: protein amount, particle number, lipid amount, expressed per volume of initial fluid or number of producing cells/mass of tissue | | | | EVs number/ mass or volume or cell number | | |
|  |  |  |  | Protein amount/ mass or volume or cell number (co-isolates) | | |
|  |  |  |  | Lipid amount/ mass or volume or cell number | | |
| Ratio of the 2 quantification figures | | | | Ratio of 2 quantification methods (normalize for protein co-isolated) | | |
| **Bulk EV characterization (Section 4-b, Table 3)** | | | | | | |
| Marker detection method | | | | Marker detection method (e.g. qPCR, WB, FC) | | |
| Transmembrane or GPI-anchored protein localized in cells at plasma membrane or endosomes + Cytosolic protein with membrane-binding or - association capacity | | | | Transmembrane or GPI-anchored protein localized in cells at plasma membrane or endosomes | | |
|  |  |  |  | Cytosolic protein with membrane-binding | | |
|  |  |  |  | Cytosolic protein with association capacity (co-isolated structure) | | |
| Assessment of presence/absence of expected contaminants (At least one each of the three categories above) | | | | / | | |
| Presence of proteins associated with compartments other than plasma membrane or endosomes | | | | / | | |
| Presence of soluble secreted proteins and their likely transmembrane ligands | | | | / | | |
| Topology of the relevant functional components (Section 4-d) | | | | / | | |
| **Single EV characterization (Section 4-c)** | | | | | | |
| Images of single EVs by wide-field and close-up: e.g. electron microscopy, scanning probe microscopy, super-resolution fluorescence microscopy | | | | EVs imaging (TEM, cryo-EM) | | |
| Non-image-based method analysing large numbers of single EVs: NTA, TRPS, FCS, high-resolution flow cytometry, multi-angle light-scattering, Raman spectroscopy, etc. | | | | EVs analysis (e.g. NTA, TRPS, hrFC) | | |
| **5 – EV Functional studies** | | | | | | |
| **EV recipient model** | | | | | | |
| ***in vitro*** | ***ex vivo*** | | | | ***in vivo*** | |
| Model species (incl. strain for rodents; breed for large animals) | | | | | | |
| Model sex | | | | | | |
| Model age | | | | | | |
| Model type | | | | | | |
| Model health status (e.g. macroscopical, histology, MRI) | | | | | | |
| Experimental set up (*in vitro, ex vivo, in vivo*) | | | | | | |
| Cell culture passage | - | | | | - | |
| Culture medium composition (additives, serum, other) | | | | - | | |
| Medium contaminants depletion protocol (FBS, plasma, natural matrix) | | | | - | | |
| Cell culture condition (monolayer, suspension, 3D) | Tissue culture condition (free swelling, constrained, bioreactor) | | | | - | |
| Cell density (cell/cm^2^, cell/mL) |  | | | |  | |
| Degeneration induction model (e.g. cytokines, needle puncture, enzymes) | | | | | | |
| Culture environment (% O_2_, % CO_2_, Temperature) | | | | | | |
| - | EVs administration (diffusion, intradiscal injection) | | | | EVs administration (intradiscal injection, intravenous injection) | |
| EV amount | | | | | | |
| EV treatment period | | | | | | |
| **Methodological controls** | | | | | | |
| Dose-response assessment | | | | Dose-response study | | |
| Negative control = nonconditioned medium, biofluid/ tissue from control donors, as applicable | | | | Negative control inclusion (e.g. medium, PBS, saline) | | |
| Quantitative comparison of functional activity of total fluid, vs EV-depleted fluid, vs EVs (after high recovery/low specificity separation) | | | | EV vs EV-depleted CM vs CM study | | |
| Quantitative comparison of functional activity of EVs vs other EPs/fractions after low recovery/high specificity separation | | | | EV vs EP study | | |
| Quantitative comparison of activity of EV subtypes (if subtype-specific function claimed) | | | | / | | |
| Extent of functional activity in the absence of contact between EV donor and EV recipient | | | | / | | |
| **Analysis** | | | | | | |
| Gene expression (e.g. qPCR) | | | | | | |
| Protein expression (e.g. (ELISA, WB) | | | | | | |
| Biochemical analysis | | | | | | |
| Histological analysis (e.g. IHC, IF) | | | | | | |
| - | | Radiological analysis | | | | |
| - | | Biomechanical analysis | | | | |
| - | | - | | | | Pain assessment |
| **EV-mediated effect** | | | | | | |
| EV effect (general brief description) | | | | | | |
| **6 - Reporting** | | | | | | |
| Submission of data (proteomic, sequencing, other) to relevant public, curated databases, or open-access repositories | | | |  | | |
| Temper EV-specific claims when MISEV requirements cannot be entirely satisfied (Section 6-b) | | | |  | | |
| Submission of methodologic details to EV-TRACK (evtrack.org) with EV-TRACK number provided (strongly encouraged) | | | |  | | |
| Data submission to EV-specific databases (e.g., EVpedia, Vesiclepedia, exRNA atlas) | | | |  | | |

***Legend:*** *Categories highlighted in yellow refer to relevant information to the IVD field. Extracellular vesicles (EVs), extracellular proteins (EPs), magnetic resonance imaging (MRI), fetal bovine serum (FBS), conditioned medium (CM), tangential flow filtration (TFF), quantitative poly chain reaction (qPCR), western blot (WB), flowcytometry (FC), transmission electron microscopy (TEM), cryo-electron microscopy (cryo-EM), nanoparticle tracking analysis (NTA), transmission resistive pulse sensing (TRPS), high-resolution flow cytometry (hrFC),* *enzyme-linked immunosorbent assay (ELISA), western blot (WB), immunohistochemistry (IHC), immunofluorescence (IF).*

### **Supplementary table 4** – List of 3 new articles (published between 2024-2025) excluded from the EV-idence analysis (*section 8*).

| **PMID** | **First Author** | **Journal** | **Publication Year** |
| --- | --- | --- | --- |
| 38382680 | Wang N | Exp Gerontol | 2024 |
| 40636356 | Zhao J | Front Med (Lausanne) | 2025 |
| 40769284 | Zhang X | Exp Gerontol | 2025 |

### **Supplementary table 5** – List of 50 new articles (published between 2024-2025) included in the EV-idence analysis (*section 8*).

| **PMID** | **First Author** | **Journal** | **Publication Year** |
| --- | --- | --- | --- |
| 38222813 | Tilotta V | JOR Spine | 2024 |
| 38362337 | Chen X | Regen Ther | 2024 |
| 38373669 | Tao X | Cell Signal | 2024 |
| 38416190 | Ambrosio L | Eur Spine J | 2024 |
| 38449582 | Aydemir E | J Neurol Surg B Skull Base | 2023 |
| 38515611 | Hu S | Bioact Mater | 2024 |
| 38583365 | Tang SN | Biomaterials | 2024 |
| 38593507 | Ma S | Int Immunopharmacol | 2024 |
| 38790015 | Lin Z | J Nanobiotechnology | 2024 |
| 38816771 | Fan C | J Nanobiotechnology | 2024 |
| 38841339 | Jia S | J Orthop Translat | 2024 |
| 38952714 | Zhang K | Biomater Res | 2024 |
| 38973294 | Jin Y | Adv Sci (Weinh) | 2024 |
| 38982049 | Zhang W | Nat Commun | 2024 |
| 38997667 | Qian G | BMC Musculoskelet Disord | 2024 |
| 39060654 | Peng S | Tissue Eng Regen Med | 2024 |
| 39079050 | Yu XJ | ACS Biomater Sci Eng | 2024 |
| 39085827 | Shi P | J Nanobiotechnology | 2024 |
| 39149595 | Zhao R | Bioact Mater | 2024 |
| 39212824 | Su KK | Stem Cell Rev Rep | 2024 |
| 39358812 | Wang L | Spine (Phila Pa 1976) | 2025 |
| 39507593 | Li L | JOR Spine | 2024 |
| 39550843 | Li Q | Int Immunopharmacol | 2024 |
| 39555665 | Zhan J | Adv Healthc Mater | 2025 |
| 39881783 | van Maanen JC | JOR Spine | 2025 |
| 39898539 | Li Q | Mol Pharm | 2025 |
| 39949490 | Martinez-Zalbidea I | Cell Mol Bioeng | 2025 |
| 39983927 | Chen J | J Control Release | 2025 |
| 39986487 | Chen C | Free Radic Biol Med | 2025 |
| 39990291 | Zhang S | Int J Nanomedicine | 2025 |
| 40027872 | Zhao R | Int J Nanomedicine | 2025 |
| 40052562 | Zhang H | Mol Med Rep | 2025 |
| 40075068 | Ma W | Bone Res | 2025 |
| 40092079 | Li C | Am J Transl Res | 2025 |
| 40118349 | Bian Z | Free Radic Biol Med | 2025 |
| 40169595 | Wang W | Nat Commun | 2025 |
| 40177564 | Chen X | Acta Pharm Sin B | 2025 |
| 40206196 | Zhao Y | Bioact Mater | 2025 |
| 40213661 | Li Y | Theranostics | 2025 |
| 40213695 | Tian Z | Front Pharmacol | 2025 |
| 40316061 | Gao X | Free Radic Biol Med | 2025 |
| 40384172 | Zhao K | Adv Healthc Mater | 2025 |
| 40440285 | Samanta A | PLoS One | 2025 |
| 40476182 | Dong Z | Stem Cells Int | 2025 |
| 40600527 | Zhang W | Curr Mol Med | 2025 |
| 40619582 | Xiang Q | Adv Sci (Weinh) | 2025 |
| 40620323 | Zhao X | Regen Biomater | 2025 |
| 40774008 | Feng J | Phytomedicine | 2025 |
| 40831373 | Shi P | J Extracell Vesicles | 2025 |
| 40842947 | Tilotta V | JOR Spine | 2025 |

### **Supplementary table 6** – Summary table of the average reporting percentages regarding the information on EV production from cell culture**.**

|  | ***Cell culture information***  ***Total = 81 studies*** | **Applicable** | **% Reported** |
| --- | --- | --- | --- |
| **Cell donor characteristics** | **Donor species** *(incl. strain for rodents; breed for large animals)* | 81 | 97.5% |
|  | **Donor sex** | 81 | 35.8% |
|  | **Donor age** | 81 | 49.4% |
|  | **Donor cell type** | 81 | 100% |
|  | **Donor health status** (*e.g. macroscopical, histology, MRI*) | 81 | 64.2% |
|  | **Cell culture passage** | 81 | 51.9% |
|  | **Mycoplasma test** *(cell line)* | 18 | 5.6% |
| **Cell culture conditions** | **Culture medium composition** *(additives, serum, other)* | 81 | 96.3% |
|  | **Medium contaminants depletion protocol** *(FBS, plasma, natural matrix) * ”* | 48 | 29.2% |
|  | **Culture medium volume** *(cells/mL)* | 81 | 17.3% |
|  | **Cell density** *(cells/cm2)* | 81 | 53.1% |
|  | **% Live/dead cells** | 81 | 4.9% |
|  | **Culture period** | 81 | 82.7% |
|  | **CM harvest frequency** | 81 | 84.0% |
|  | **Cell treatment** *(stimuli: e.g. cytokines) ** | 12 | 100% |
|  | **Culture condition** *(monolayer, 3D, suspension)* | 81 | 95.1% |
|  | **Culture environment** *(% O_2_, % CO_2_, Temperature)* | 81 | 70.4% |
|  | ***Average EV source cell culture info reporting percentage*** | | ***61.0%*** |

***Note:*** *Magnetic resonance imaging (MRI), fetal bovine serum (FBS), conditioned medium (CM).* *For each information category, number of applicable studies and percentage of studies that reported the information are listed. “ Minimal EVs’ research requirement (MISEV guidelines). * Optional information.*

### **Supplementary table 7** - Summary table of the average reporting percentages regarding the information on EV production from biofluids or tissue culture.

|  | ***Biofluids or tissue culture information***  ***Total = 9 studies*** | **Applicable** | **% reported** |
| --- | --- | --- | --- |
| **Tissue donor characteristics** | **Donor species** *(incl. strain for rodents; breed for large animals)* | 9 | 100% |
|  | **Donor sex** | 9 | 44.4% |
|  | **Donor age** | 9 | 66.7% |
|  | **Sample type** *(tissue, biofluid)* | 9 | 100% |
|  | **Donor health status** (*e.g. macroscopical, histology, MRI*) | 9 | 66.7% |
| **Tissue culture conditions** | **Culture medium composition** *(additives, serum, other)* | 5 | 83.3% |
|  | **Medium contaminants depletion protocol** *(FBS, plasma, natural matrix) * “* | 1 | 0.0% |
|  | **Collected volume or volume/mass per donor** | 9 | 66.7% |
|  | **Collection site** *(sampling location)* | 5 | 80.0% |
|  | **Time and temperature of biofluid or tissue collection** *(fresh, cadaveric)* | 9 | 88.9% |
|  | **Total pooled sample volume or mass** | 9 | 44.4% |
|  | **% live/dead cells** | 5 | 0.0% |
|  | **Culture period** | 5 | 80.0% |
|  | **CM or biofluid harvest frequency** | 9 | 88.9% |
|  | **Sample treatment** *(stimuli: e.g. cytokines) ** | 1 | 100% |
|  | **Culture condition** *(free swelling, constrained, bioreactor)* | 5 | 100% |
|  | **Culture environment** *(% O_2_, % CO_2_, Temperature)* | 9 | 55.6% |
|  | **Tissue treatment method for direct EVs isolation** *(enzymatic activity or mg/ml, chemical-physical) ** | 0 | - |
|  | **Treatment time *** | 0 | - |
|  | **Treatment temperature *** | 0 | - |
|  | ***Average EV source tissue culture info reporting percentage*** | | ***68.4%*** |

***Note:*** *Magnetic resonance imaging (MRI), fetal bovine serum (FBS), conditioned medium (CM). For each information category, number of applicable studies and percentage of studies that reported the information are listed. “ Minimal EVs’ research requirement. * Optional information.*

### **Supplementary table 8** - Summary table of the average reporting percentages of storage of biofluids, conditioned medium or isolated EVs.

| ***Storage information***  ***Total = 90 studies*** | **Applicable** | **% reported** |
| --- | --- | --- |
| **CM or biofluid or tissue storage temperature** | 90 | 12.2% |
| **CM or biofluid storage vessel** *(low-binding tubes)* | 90 | 0.0% |
| **EVs storage method** *(e.g. frozen, lyophilized)* | 89 | 73.0% |
| **EVs storage temperature** | 89 | 49.4% |
| **EVs storage vessel** *(low-binding tubes)* | 89 | 0.0% |
| ***Average storage information reporting percentage*** | | **26.9%** |

***Note:*** *Conditioned medium (CM), extracellular vesicles (EVs). For each information category, number of applicable studies and percentage of studies that reported the information are listed.*

### **Supplementary table 9** - Summary table of the reporting percentages regarding the information on isolation and concentration methods used to enrich for EVs.

| ***EV isolation and concentration information***  ***Total = 90 studies*** | **Applicable** | **% reported** |
| --- | --- | --- |
| **EV recovery method** *(ultracentrifugation, density gradient, chromatography, precipitation, filtration, antibody-affinity)* | 90 | 96.7% |
| **Method details** | 90 | 96.7% |
| **Concentration method** *(e.g. centrifugal filter tube, tangential-flow filtration) ** | 6 | 100% |
| **Matrix or sample washing method *** | 42 | 100% |
| ***Average EV isolation information reporting percentage*** | | ***98.3%*** |

***Note:*** *Extracellular vesicle (EV). For each information category, number of applicable studies and percentage of studies that reported the information are listed. * Optional information.*

**Supplementary table 10** – Frequency of each method used as primary technique or in combination with a sequential method to further enrich for EVs.

| ***Isolation methods combinations***  ***Total = 90 studies*** | ***Recovery vs specificity*** | ***First method*** | ***Follow-up method*** |
| --- | --- | --- | --- |
| **Differential ultracentrifugation** | intermediate | 70 | 0 |
| **Filtration** | high vs low | 10 | 0 |
| **Precipitation** | high vs low | 7 | 0 |
| **Density gradient** | low vs high | 0 | 3 |
| **Size exclusion chromatography** | intermediate | 0 | 1 |
| **Immuno-isolation** | low vs high | 0 | 0 |
| **Not reported** | - | 3 | 0 |

***Note:*** *Follow-up method refers to the EV enrichment technique used in sequential combination with any of the possible first method.*

### **Supplementary table 11** - Summary table of the reporting percentages for quantification methodology of isolated EVs.

| ***EV quantification information***  ***Total = 90 studies*** | **Applicable** | **% reported** |
| --- | --- | --- |
| **Fluid volume or cell number or tissue mass used to isolate EVs** | 90 | 25.6% |
| **EVs number/ mass or volume or cell number** | 90 | 24.4% |
| **Protein amount/ mass or volume or cell number** *(co-isolates)* | 90 | 16.7% |
| **Lipid amount/ mass or volume or cell number** | 90 | 1.1% |
| **Ratio of 2 quantification methods** *(co-isolated protein normalization)* | 90 | 1.1% |
| ***Average EV quantification info reporting percentage*** | | ***13.8%*** |

***Note:*** *for each information category, number of applicable studies and percentage of studies that reported the information are listed.*

### **Supplementary table 12** - Summary table of the reporting percentages regarding characterization of isolated EVs.

| ***EV characterization information***  ***Total = 90 studies*** | **Applicable** | **% reported** |
| --- | --- | --- |
| **Marker detection method** *(e.g. qPCR, WB, FC)* | 90 | 85.6% |
| **Transmembrane or GPI-anchored protein derived from plasma membrane or endosomal membranes** | 90 | 74.4% |
| **Cytosolic protein** | 90 | 74.4% |
| **Co-isolates** | 90 | 41.1% |
| **Average reporting bulk EV characterization percentage** | | **68.9%** |
| **EVs imaging** *(TEM, cryo-EM)* | 90 | 90.0% |
| **EVs analysis** *(e.g. NTA, TRPS, hrFC)* | 90 | 77.8% |
| **Average reporting single EV characterization percentage** | | **83.9%** |
| ***Average EV characterization info reporting percentage*** | | ***73.9%*** |

***Note:*** *for each information category, number of applicable studies and percentage of studies that reported the information are listed. Quantitative poly chain reaction (qPCR), western blot (WB), flowcytometry (FC), transmission electron microscopy (TEM), cryo-electron microscopy (cryo-EM), nanoparticle tracking analysis (NTA), transmission resistive pulse sensing (TRPS), high-resolution flow cytometry (hrFC).*

### **Supplementary table 13** - Summary table of the average reporting percentages for EVs functional studies *in vitro*.

|  | ***EV functional studies in vitro***  ***Total = 82 studies*** | **Applicable** | **% reported** |
| --- | --- | --- | --- |
| **Cell model characteristics** | **Model species** *(incl. strain for rodents; breed for large animals)* | 82 | 98.8% |
|  | **Model sex** | 82 | 32.9% |
|  | **Model age** | 82 | 52.4% |
|  | **Model cell type** | 82 | 97.6% |
|  | **Model health status** (*e.g. macroscopical, histology, MRI*) | 82 | 67.1% |
|  | **Cell culture passage** | 82 | 59.8% |
| **Cell culture conditions** | **Culture medium composition** *(additives, serum, other)* | 82 | 90.2% |
|  | **Medium contaminants depletion protocol** *(FBS, plasma, natural matrix, purity additives) “* | 10 | 10.0% |
|  | **Cell culture model** *(monolayer, 3D, suspension)* | 82 | 100% |
|  | **Cell density** *(cell/cm^2^)* | 82 | 63.4% |
|  | **Degeneration induction model** *(stressors: e.g. cytokines) ** | 55 | 100% |
|  | **Culture environment** *(% O_2_, % CO_2_, Temperature)* | 82 | 73.2% |
| **EV treatment** | **EVs amount** | 82 | 80.5% |
|  | **EVs treatment period** | 82 | 92.7% |
|  | ***Average reporting in vitro model system*** | | ***72.8%*** |
| **Methodological controls** | **EV dose-response study** | 82 | 28.0% |
|  | **Negative control** *(e.g. medium)* **“** | 82 | 97.6% |
|  | **EVs vs EV-depleted vs CM study “** | 82 | 12.2% |
|  | **EVs vs EPs study** | 82 | 8.5% |
|  | ***Average reporting methodological controls*** | | ***36.6%*** |
| **Analysis** | **Gene expression** *(e.g. qPCR)* | 82 | 79.3% |
|  | **Protein expression** *(e.g. ELISA, WB)* | 82 | 84.1% |
|  | **Biochemical analysis** | 82 | 81.7% |
|  | **Histological analysis** *(e.g. IHC, IF)* | 82 | 86.6% |
|  | ***Average reporting in vitro analysis*** | | ***82.9%*** |
|  | ***Average reporting percentage for in vitro EV functional studies*** | | ***68.0%*** |

**LEGEND:** Magnetic resonance imaging (MRI), fetal bovine serum (FBS), conditioned medium (CM), extracellular vesicles (EVs), extracellular proteins (EPs), quantitative poly chain reaction (qPCR), enzyme-linked immunosorbent assay (ELISA), western blot (WB), immunohistochemistry (IHC), immunofluorescence (IF). “Minimal EVs’ research requirement. * Optional information

### **Supplementary table 14** - Summary table of the average reporting percentages for EVs functional studies *ex vivo*.

|  | ***EV functional studies ex vivo***  ***Total = 3 studies*** | **Applicable** | **% reported** |
| --- | --- | --- | --- |
| **Explant model characteristics** | **Model species** *(incl. strain for rodents; breed for large animals)* | 3 | 100% |
|  | **Model sex** | 3 | 100% |
|  | **Model age** | 3 | 100% |
|  | **Model type** *(sampling location; whole IVD, NP explant)* | 3 | 66.7% |
|  | **Model health status** (*e.g. macroscopical, histology, MRI*) | 3 | 0.0% |
| **Explant culture conditions** | **Culture medium composition** *(additives, serum, other)* | 3 | 100% |
|  | **Medium contaminants depletion protocol** *(FBS, plasma, natural matrix, purity additives) “* | 3 | 0.0% |
|  | **Culture model** *(free swelling, constrained, bioreactor)* | 3 | 100% |
|  | **Degeneration induction model** *(stressors: e.g. cytokines, enzymes) ** | 2 | 100% |
|  | **Culture environment** *(% O_2_, % CO_2_, Temperature)* | 3 | 66.7% |
| **EV treatment** | **EV administration** *(diffusion, intradiscal injection)* | 3 | 100% |
|  | **EVs amount** | 3 | 100% |
|  | **EVs treatment period** | 3 | 100% |
|  | ***Average ex vivo model reporting percentage*** | | ***79.5%*** |
| **Methodological controls** | **EV dose-response study** | 3 | 0.0% |
|  | **Negative control** *(e.g. saline, PBS, medium)* **“** | 3 | 100% |
|  | **EVs vs EV-depleted vs CM study “** | 3 | 0.0% |
|  | **EVs vs EPs study** | 3 | 0.0% |
|  | ***Average reporting methodological controls*** | | ***25.0%*** |
| **Analysis** | **Gene expression** *(e.g. qPCR)* | 3 | 0.0% |
|  | **Protein expression** *(e.g. ELISA, WB)* | 3 | 0.0% |
|  | **Biochemical analysis** | 3 | 66.7% |
|  | **Histological analysis** *(e.g. IHC, IF)* | 3 | 66.7% |
|  | **Radiological analysis** | 3 | 100% |
|  | ***Average reporting ex vivo analysis*** | | ***46.7%*** |
|  | ***Average reporting percentage for ex vivo EV functional studies*** | | ***62.1%*** |

**LEGEND:** Magnetic resonance imaging (MRI), fetal bovine serum (FBS), conditioned medium (CM), extracellular vesicles (EVs), extracellular proteins (EPs), phosphate-buffered saline (PBS), quantitative poly chain reaction (qPCR), enzyme-linked immunosorbent assay (ELISA), western blot (WB), immunohistochemistry (IHC), immunofluorescence (IF). **“**Minimal EVs’ research requirement. * Optional information

### **Supplementary table 15** - Summary table of the average reporting percentages for EVs functional studies *in vivo*.

|  | ***EV functional studies in vivo***  ***Total = 43 studies*** | **Applicable** | **% reported** |
| --- | --- | --- | --- |
| **Animal model characteristics** | **Model species** *(incl. strain for rodents; breed for large animals)* | 43 | 100% |
|  | **Model sex** | 43 | 69.8% |
|  | **Model age** | 43 | 86.0% |
|  | **Model health status** (*e.g. macroscopical, histology, MRI*) | 43 | 44.2% |
| **Experimental conditions** | **Anaesthesia procedure** | 43 | 90.7% |
|  | **Degeneration induction model** *(stressors: e.g. needle puncture) ** | 43 | 100% |
|  | **Animal environment** *(e.g. food, water, day-night cycles)* | 43 | 30.2% |
| **EV treatment** | **Injection model** *(intradiscal injection, intravenous injection)* | 43 | 100% |
|  | **Injection location** | 43 | 97.7% |
|  | **EVs amount** | 43 | 97.7% |
|  | **EVs treatment period** | 43 | 100% |
|  | ***Average in vivo model reporting percentage*** | | ***83.3%*** |
| **Methodological controls** | **EV dose-response study** | 43 | 0.0% |
|  | **Negative control** *(e.g. saline, PBS, medium)* **“** | 43 | 100% |
|  | **EVs vs EV-depleted vs CM study “** | 43 | 4.7% |
|  | **EVs vs EPs study** | 43 | 0.0% |
|  | ***Average reporting percentage methodological controls*** | | ***26.2%*** |
| **Analysis** | **Gene expression** *(e.g. qPCR)* | 43 | 23.3% |
|  | **Protein expression** *(e.g. ELISA, WB)* | 43 | 51.2% |
|  | **Biochemical analysis** | 43 | 39.5% |
|  | **Histological analysis** *(e.g. IHC, IF)* | 43 | 97.7% |
|  | **Radiological analysis** | 43 | 83.7% |
|  | **Biomechanical analysis** | 43 | 9.3% |
|  | **Pain analysis** | 43 | 0.0% |
|  | ***Average reporting percentage in vivo analysis*** | | ***43.5%*** |
|  | ***Average reporting percentage in vivo EV functional studies*** | | ***60.3%*** |

**LEGEND:** Magnetic resonance imaging (MRI), phosphate-buffered saline (PBS), conditioned medium (CM), extracellular vesicles (EVs), extracellular proteins (EPs), quantitative poly chain reaction (qPCR), enzyme-linked immunosorbent assay (ELISA), western blot (WB), immunohistochemistry (IHC), immunofluorescence (IF). “Minimal EVs’ research requirement. * Optional information

**Supplementary table 16** – Summary table of the average information reporting percentages of the 133 EV studies per category.

| ***SECTIONS*** | ***MACRO-CATEGORIES*** | **Average % information reported** | | |
| --- | --- | --- | --- | --- |
| **EV characterization** | Nomenclature | 36.7% | | |
|  | Cell Culture | 61.0% | | |
|  | Biofluids or Tissue Culture | 68.4% | | |
|  | Storage | 26.9% | | |
|  | EV isolation | 98.3% | | |
|  | EV quantification | 13.8% | | |
|  | Bulk EV characterization | 68.9% | | |
|  | Single EV characterization | 83.9% | | |
|  |  | ***in vitro*** | ***ex vivo*** | ***in vivo*** |
| **EV functional studies** | EV recipient model | 72.8% | 79.5% | 83.3% |
|  | Methodological controls | 36.6% | 25.0% | 26.2% |
|  | Analysis | 82.9% | 46.7% | 43.5% |

**Supplementary table 17** – Summary table of the studies reporting the minimal essential information (EV-depletion serum protocol) and including the methodological controls (EV-depleted medium or conditioned medium).

| **Article** | **EV characterization study** | | | **EV functional study** | | | | |
| --- | --- | --- | --- | --- | --- | --- | --- | --- |
| **Reference** | **EV source** | **Culture medium** | **Serum EV-depletion protocol** | **Set up** | **Culture medium** | **Serum EV-depletion protocol** | **Methodological controls** | **EV-depletion protocol** |
| *Lan WR et al. (2019)* | Cell culture | 10% EV-depleted serum-containing culture medium | 120,000 g 90 min | *in vitro* | Serum-free culture medium | N.A. | EV-depleted medium | 120,000 × g 70 min |
| *González-Cubero E. et al. (2022)* | Cell culture | Serum-free culture medium | N.A. | *in vitro* | Serum-free culture medium | N.A. | Conditioned medium and EV-depleted medium | 100,000 x g 70 min twice |
|  |  |  |  | *in vitro* | Serum-free culture medium | N.A. | Conditioned medium and EV-depleted medium | 100,000 x g 70 min twice |
| *Qian J et al. (2022)* | Biofluid | N.A. | N.A. | *in vivo* | N. A | N.A. | whole PRP | N.A. |
| *Dai Z et al. (2023)* | Biofluid | N.A. | N.A. | *in vitro* | Serum-free  culture medium | N.A. | whole PRP | N.A. |
|  |  |  |  | *in vivo* | N.A. | N.A. | whole PRP | N.A. |
| *van Maanen JC et al. (2025)* | Tissue culture | Serum-free culture medium | N.A. | *in vitro* | Serum-free culture medium | N.A. | EV-depleted medium | 100,000 x g 15–18 h |
|  |  |  |  | *in vivo* | Serum-free culture medium | N.A. | EV-depleted medium | 100,000 x g 15–18 h |

**Note:** articles ordered by publication year. Not applicable (N.A.).
